# Full‐Wave Harmonic Balance Framework for Dispersive Time‐Varying Photonic Structures

**DOI:** 10.1002/nap2.70361

**Published:** 2026-11-13

**Authors:** Mohammad R. Tavakol, Wenshan Cai

**Affiliations:** ^1^ School of Electrical and Computer Engineering Georgia Institute of Technology Atlanta Georgia USA; ^2^ School of Materials Science and Engineering Georgia Institute of Technology Atlanta Georgia USA

**Keywords:** dispersion, epsilon‐near‐zero materials, Floquet harmonics, metasurfaces, time‐varying photonics

## Abstract

Time‐varying photonic structures redistribute electromagnetic energy among Floquet harmonics, enabling frequency conversion, nonreciprocity, parametric gain, and dynamic wavefront control. Accurate modeling of realistic platforms remains challenging when temporal modulation occurs in strongly dispersive materials, because each harmonic experiences a distinct material response while remaining coupled to all others through the modulation. This work introduces a full‐wave harmonic‐balance framework for dispersive time‐varying photonic structures. The formulation solves the steady‐state Floquet response in the frequency domain by representing modulated components as induced secondary sources: volumetric polarization densities for bulk media and surface current densities for conductive sheets. Material dispersion and radiation operators are evaluated at each harmonic frequency, whereas temporal modulation enters as off‐diagonal convolutional coupling in Floquet space. The framework is validated for a parametrically pumped rolled graphene cylinder, where numerical results agree with an analytical Floquet scattering solution and resolve harmonic‐specific scattering, absorption, and near fields. It is further applied to an ITO‐based epsilon‐near‐zero (ENZ) space‐time metasurface, enabling shape optimization of a reflective device that converts an optical input into sidebands and redirects them into selected spatial diffraction orders. The approach establishes a general platform for modeling and designing dispersive active photonic structures with harmonic‐resolved field, power, absorption, and diffraction observables.

## Graphical abstract

A harmonic‐balance solver enables accurate modeling and design of time‐varying photonic structures with dispersive materials. By representing temporal modulation as coupled secondary sources across multiple frequencies, the method captures frequency conversion, scattering, absorption, and diffraction. Validated with graphene and an epsilon‐near‐zero metasurface, the approach provides a versatile framework for designing dynamic photonic devices.

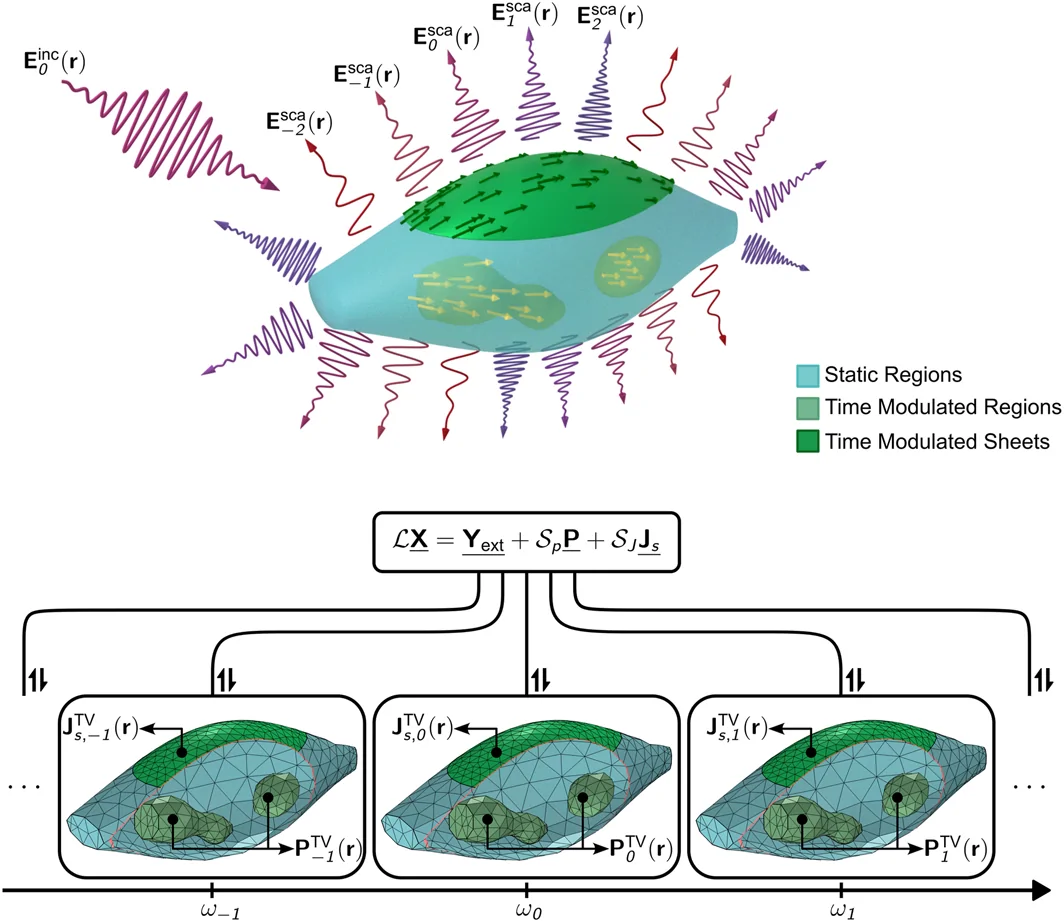

## 1 Introduction

Time‐varying photonic media extend conventional wave engineering by adding time as an externally controlled degree of freedom. In a static structure, temporal translation symmetry enforces conservation of optical frequency, and spatial structuring can only redistribute the incident field among spatial modes at the same frequency. When a material parameter is modulated in time, this symmetry is broken: an incident wave at $\omega_{0}$ can exchange energy with the modulation and generate Floquet harmonics $\omega_{n}=\omega_{0}+n\Omega$, where Ω is the modulation frequency [1]. If the modulation also varies in space, the generated harmonics can simultaneously acquire transverse momentum, producing space–time diffraction channels indexed by both temporal and spatial orders. This additional synthetic dimension has enabled new routes to magnet‐free nonreciprocity, frequency conversion, parametric amplification, harmonic‐domain beam steering, and dynamic wavefront control [2, 3, 4, 5, 6, 7].

Early work on time‐gradient and space–time‐gradient metasurfaces showed that properly designed temporal and transverse phase gradients can generalize Snell's law by allowing both momentum and energy exchange with an ultrathin surface, thereby enabling Lorentz nonreciprocity without magnetic bias [3, 4]. Subsequent metasurface platforms demonstrated that parametric sidebands are not merely parasitic by‐products of modulation, but useful output channels whose phase, amplitude, and directionality can be engineered. Time‐modulated Huygens' metadevices used independent electric and magnetic modulation to control sideband radiation direction and achieve efficient parametric wavefront control [5], whereas rigorous space–time coupled‐wave and free‐space N‐path formulations provided design tools for patterned, spatially discretized, and programmable space–time metasurfaces [6, 7]. More recently, temporally and spatiotemporally modulated metasurfaces have been used to realize optical frequency conversion, transmissive time‐modulated nonreciprocity, ultrafast all‐optical beam steering, and multifrequency beam shaping on conformal apertures [8, 9, 10, 11]. Even a single‐period traveling pulse modulation has been theoretically shown to mimic the selective photonic transitions of continuous periodic driving when combined with dispersion‐engineered metasurfaces, enabling strong optical nonreciprocity [12].

Beyond wavefront control, temporal modulation can change the energetic character of a photonic system. Photonic time crystals and time‐varying resonant media can open momentum band gaps, support parametrically amplifying solutions, and convert energy supplied by the modulation into electromagnetic radiation [13, 14, 15, 16]. Such momentum‐gap physics was recently observed experimentally in a terahertz plasmonic metamaterial where near‐unity sub‐cycle modulation of the carrier effective mass produced parametric gain and reduced plasmonic losses [17]. Such effects have led to proposals and demonstrations of coherent perfect absorption and amplification in time‐varying epsilon‐near‐zero (ENZ) media, all‐optical polarization control through time‐varying low‐index films, and nonreciprocal negative refraction enabled by photonic time crystals [16, 18, 19]. At the same time, generalized space–time structures such as moving interfaces, slabs, crystals, gradients, and wedges illustrate that even simple time‐dependent geometries can generate rich cascades of frequency‐converted scattering channels [20, 21]. These advances show that the central design variables in time‐varying photonics are no longer only geometry and static material dispersion, but also the spectrum, phase, and spatial distribution of the modulation itself.

Accurately modeling such systems is challenging because the strongest practical time‐varying platforms are often highly dispersive. Transparent conducting oxides, ENZ films, graphene, and resonant meta‐atoms derive their large modulation sensitivity from free‐carrier or resonant material responses, whose permittivity or conductivity changes rapidly with frequency. In these systems, temporal modulation cannot be treated as a simple instantaneous perturbation of a frequency‐independent material parameter. Causality requires a response with memory, and in a linear time‐varying medium, the polarization at time t depends on both the observation time and the earlier excitation history. This has motivated generalized Kramers–Kronig relations, temporal‐complex‐polarizability formalisms, and causality‐aware descriptions of dispersive time‐varying media [22, 23, 24]. It has also exposed important numerical pitfalls: solvers that implicitly apply time‐static update rules can violate the continuity of the electric displacement field and give incorrect results when the material parameters vary in time [25].

Several computational and semi‐analytical frameworks have been developed to address different parts of this problem. Time‐domain methods, including FDTD‐based and GSTC‐FDTD approaches, can represent broadband dynamics and surface susceptibilities, but they may require long simulations to reach the steady‐state multi‐harmonic response and must be implemented with care for causal, dispersive, time‐varying media [20, 26]. Coupled‐wave, transfer‐matrix, and modal methods provide efficient descriptions for periodic or canonical geometries, and Floquet–Mie theory gives a rigorous solution for time‐varying dispersive spheres [6, 13]. These methods have been instrumental in clarifying the physics of space–time diffraction, temporal band gaps, and parametric gain. However, many practical photonic structures combine features that are difficult to capture within a single specialized formalism: complex two‐ or three‐dimensional geometries, strongly dispersive volumetric media, modulated zero‐thickness sheets, multiple coupled Floquet harmonics, and harmonic‐resolved near fields, powers, and absorption.

Here, we present a full‐wave harmonic‐balance framework for dispersive time‐varying photonic structures. The method solves directly for the steady‐state Floquet response in the frequency domain, avoiding time marching while retaining the coupling among all retained harmonics. The key idea is to represent the time‐varying components through induced secondary sources: volumetric polarization densities for modulated dispersive media and surface current densities for modulated material sheets. Temporal modulation then appears as a convolution in Floquet space, producing off‐diagonal coupling between harmonic subproblems, whereas material dispersion, radiation operators, and source‐injection operators are evaluated at the physical frequency of each harmonic. This formulation treats time‐varying volumetric media and time‐varying conductive sheets within the same algebraic structure, supports arbitrary temporal modulation spectra through the Fourier coefficients of the material parameters, and yields harmonic‐resolved observables such as scattered power, absorption, diffraction amplitudes, and near‐field distributions. This capability is particularly important for dispersive platforms where the response at frequencies shifted from the carrier by integer multiples of the modulation frequency cannot be inferred from a single static material value at the carrier frequency. Instead, each generated harmonic must be solved with its own material response while remaining coupled to the other harmonics through the modulation‐induced secondary sources. In this way, the framework combines the physical transparency of harmonic‐balance and multi‐frequency frequency‐domain approaches [27, 28] with the geometric flexibility of full‐wave finite‐element modeling.

We demonstrate the framework with two representative examples that establish its applicability to both surface‐ and volume‐modulated dispersive platforms. First, we analyze a parametrically pumped rolled graphene cylinder whose Fermi level is modulated in time. This example tests the surface‐current version of the formulation in a strongly dispersive THz platform and in a degenerate parametric‐amplification regime where $\Omega =2\omega_{0}$. The harmonic‐balance results are compared with an analytical Floquet scattering solution derived in Supporting Information S1, showing excellent agreement for harmonic‐resolved scattering and absorption. Second, we design a reflective optical space–time metasurface consisting of a binary ${\text{TiO}}_{2}$ grating on a time‐modulated ITO ENZ layer backed by a gold reflector. This example tests the volumetric‐polarization version of the framework in a dispersive Drude medium whose plasma frequency is modulated in time. The optimized metasurface converts the incident field into sideband harmonics and preferentially redirects the generated sidebands into the first spatial diffraction order, demonstrating frequency‐converting beam deflection in a realistic optical space–time platform. By solving the coupled Floquet harmonics as a single full‐wave system, the proposed harmonic‐balance framework captures the essential interplay among temporal modulation, material dispersion, spatial scattering, parametric energy exchange, and harmonic‐domain radiation.

## 2 Harmonic‐Balance Formulation

The proposed full‐wave harmonic‐balance framework is schematically summarized in Figure 1. We consider a general dispersive photonic structure containing static regions, time‐modulated volumetric media, and time‐modulated material sheets or interfaces. As illustrated in Figure 1a, the structure is illuminated by an incident field at angular frequency $\omega_{0}$, whereas the time‐varying components generate frequency‐converted scattered fields at multiple Floquet harmonics. In this representation, the action of the time‐varying elements is described through induced secondary sources: polarization densities in modulated volumetric regions and surface current densities on modulated sheets. These secondary sources provide the physical mechanism by which energy is transferred among harmonic channels.

**FIGURE 1:**
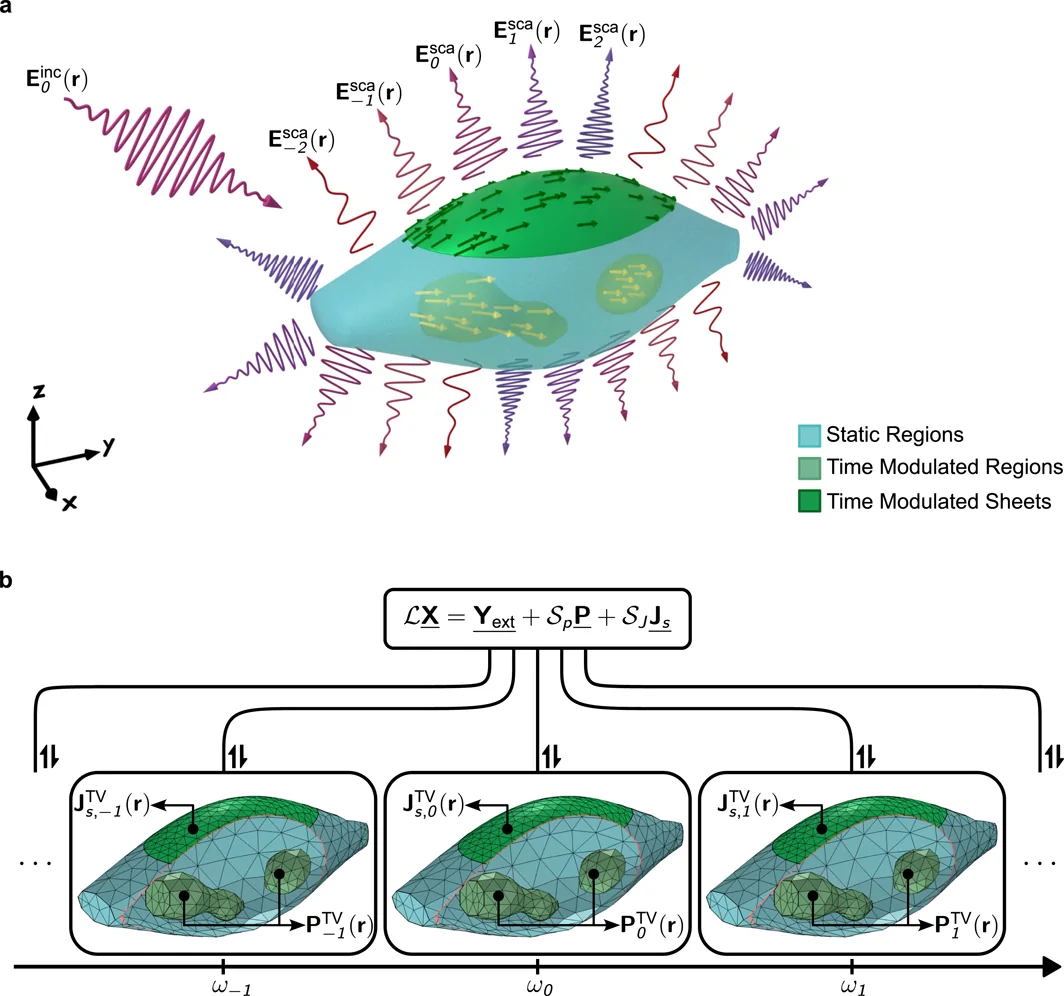
Harmonic‐balance solver via secondary‐source representation. (a) Schematic of a time‐varying photonic structure illuminated by an incident field $E_{0}^{\text{inc}}\left(r\right)$. Yellow vectors inside the light‐green regions represent induced secondary polarization sources, whereas dark‐green vectors on the green sheets denote induced surface‐current sources. The colored outgoing waves indicate multi‐harmonic scattering generated by these secondary sources. (b) Harmonic‐balance representation of the same system as a set of coupled frequency‐domain FEM subproblems. Each mesh view corresponds to one Floquet harmonic $\omega_{n}$ with the subproblems coupled through the secondary sources governed by the global multi‐harmonic system.

For a modulation frequency Ω, the generated harmonic frequencies are

(1) $$\omega_{n}=\omega_{0}+n\Omega ,$$

where n is the Floquet‐harmonic index. The central objective of the harmonic‐balance formulation is to solve for the steady‐state electromagnetic response at these coupled harmonic frequencies directly without time marching. For a time‐periodic system, any field or induced source quantity can be expanded in a truncated Floquet series as

(2) $$F\left(r,t\right)=\sum_{n=-N}^{N}F_{n}\left(r\right)e^{j\omega_{n}t},$$

where 2N+1 harmonics are retained. The unknown electromagnetic fields are collected into a multi‐harmonic vector

(3) $$\underline{X}={\left[\begin{array}{ccccc} X_{-N} & \text{⋯} & X_{0} & \text{⋯} & X_{N} \end{array}\right]}^{T},$$

where the underline denotes the vector space of retained Floquet harmonics. Each component $X_{n}$ represents the field unknowns at frequency $\omega_{n}$. Thus, the original time‐varying problem is recast as a coupled set of frequency‐domain electromagnetic problems.

The induced polarization and surface‐current sources are expanded in the same harmonic basis,

(4) $$\underline{P}={\left[\begin{array}{ccccc} P_{-N} & \text{⋯} & P_{0} & \text{⋯} & P_{N} \end{array}\right]}^{T},\;\underline{J_{s}}={\left[\begin{array}{ccccc} J_{s,-N} & \text{⋯} & J_{s,0} & \text{⋯} & J_{s,N} \end{array}\right]}^{T}.$$

In a static linear system, the response at each frequency is independent. In contrast, a time‐periodic material coefficient mixes spectral components through convolution in harmonic space. As a result, the field at one harmonic can induce polarization or surface current at other harmonics, which then radiate or scatter back into the electromagnetic system. This is the physical origin of the multi‐harmonic scattering channels shown in Figure 1a.

Using this secondary‐source representation, the multi‐harmonic electromagnetic problem can be written compactly as

(5) $$L\;\underline{X}=\underline{Y_{\text{ext}}}+S_{P}\underline{P}+S_{J}\underline{J_{s}}.$$

where L is the block frequency‐domain Maxwell operator for the static background structure, $\underline{Y_{\text{ext}}}$ is the external excitation projected into the harmonic space, and $S_{P}$ and $S_{J}$ are source‐injection operators for volumetric polarization and sheet‐current sources, respectively. Equation (5) should not be interpreted as an uncoupled driven problem, because the secondary sources $\underline{P}$ and $\underline{J_{s}}$ are not prescribed external sources. They are induced by the unknown multi‐harmonic fields through the time‐varying material response. The source‐injection blocks may depend on the harmonic frequency, for example, through $S_{P,n}\left(\omega_{n}\right)$ and $S_{J,n}\left(\omega_{n}\right)$, so that dispersive source‐to‐field mappings, radiation conditions, and sheet/source normalization factors are evaluated at the physical frequency of each harmonic. The inter‐harmonic coupling itself, however, is contained in the source‐generation relations described below.

To make the coupling explicit, consider a material or sheet parameter with a temporal Fourier expansion

(6) $$m\left(r,t\right)=\sum_{\ell =-L}^{L}m_{\ell }\left(r\right)e^{j\ell \Omega t}.$$

Multiplication of this modulation waveform by the multi‐harmonic field produces a convolution in Floquet space. Therefore, the induced secondary sources at $\omega_{n}$ generally depend on field components at neighboring or more distant harmonics,

(7) $$P_{n}=\sum_{\ell =-L}^{L}C_{n,n-\ell }^{P}X_{n-\ell },\;J_{s,n}=\sum_{\ell =-L}^{L}C_{n,n-\ell }^{J}X_{n-\ell }.$$

where $C_{n,n-\ell }^{P}$ and $C_{n,n-\ell }^{J}$ denote the volumetric and sheet source‐generation blocks associated with the ℓth modulation order; terms outside the retained harmonic window are omitted by the truncation. A single‐tone modulation produces nearest‐neighbor harmonic coupling, whereas a multi‐tone or nonsinusoidal modulation produces a block‐banded coupling matrix whose harmonic bandwidth is determined by L. This representation naturally supports arbitrary modulation spectra, including traveling‐wave, multi‐tone, and waveform‐engineered temporal modulations.

In global form, the induced‐source relation can be written as

(8) $$\underline{P}=C_{P}\underline{X},\;\underline{J_{s}}=C_{J}\underline{X},$$

or, equivalently,

(9) $$\underline{Q}=C\;\underline{X},\;\underline{Q}=\left[\begin{array}{c} \underline{P}\\ \underline{J_{s}} \end{array}\right],\;C=\left[\begin{array}{c} C_{P}\\ C_{J} \end{array}\right].$$

The operators $C_{P}$ and $C_{J}$ are generally nondiagonal in harmonic space. Their diagonal blocks describe the same‐frequency material response, whereas their off‐diagonal blocks describe frequency conversion between harmonics separated by integer multiples of Ω. The number and strength of these off‐diagonal couplings are determined by the Fourier spectrum of the temporal modulation.

Defining

(10) $$S=\left[\begin{array}{cc} S_{P} & S_{J} \end{array}\right],$$

the secondary‐source formulation becomes

(11) $$L\;\underline{X}=\underline{Y_{\text{ext}}}+S\underline{Q}.$$

Substitution of the induced‐source relations gives the closed global harmonic‐balance system,

(12) $$\left(L-S_{P}C_{P}-S_{J}C_{J}\right)\underline{X}=\underline{Y_{\text{ext}}},$$

or, in compact form,

(13) $$A_{\text{HB}}\underline{X}=\underline{Y_{\text{ext}}},\;A_{\text{HB}}=L-SC.$$

Equation (13) is the central algebraic form of the proposed framework and corresponds to the global multi‐harmonic system shown in Figure 1b. The diagonal blocks represent independent full‐wave problems at the harmonic frequencies $\omega_{n}$, whereas the off‐diagonal blocks originate from the secondary sources induced by the time‐varying components. Therefore, the full time‐varying photonic structure is solved as a single coupled system in the extended Floquet‐frequency space.

In a finite‐element implementation, each harmonic component is solved using a frequency‐domain weak form. The conventional FEM unknown vector is lifted into a harmonic‐domain vector, so that each spatial degree of freedom carries multiple harmonic coefficients. As depicted by the mesh views in Figure 1b, each harmonic subproblem retains the full spatial complexity of the electromagnetic geometry, while remaining coupled to the other harmonics through the secondary‐source terms. For 2N+1 retained harmonics, the total number of unknowns scales approximately by the same factor relative to a single‐frequency FEM problem. However, the resulting matrix has a structured block form: the diagonal blocks contain the standard full‐wave FEM operators at individual harmonic frequencies, while the off‐diagonal blocks encode coupling through the induced polarization and surface‐current sources.

The present framework is closely related in spirit to multi‐frequency frequency‐domain solvers developed for active nanophotonic devices. In particular, the MF‐FDFD algorithm of Shi et al. couples multiple frequency‐domain Maxwell systems through modulation‐induced polarization terms [28]. While the MF‐FDFD algorithm couples the Floquet harmonics through the polarization induced by an instantaneous, dispersionless permittivity modulation and can likewise be formulated on a finite‐element grid, the present framework derives its secondary sources from the time‐domain constitutive relations of dispersive modulated media and conductive sheets. As a result, each generated harmonic experiences the material response at its own physical frequency, and zero‐thickness time‐varying sheets enter the weak form directly as interface terms, two features that are essential for the dispersive graphene and epsilon‐near‐zero structures considered in this work. The present formulation generalizes this viewpoint in several directions. First, the modulation spectrum is not restricted to a single sinusoidal tone: an arbitrary number of temporal Fourier components can be included through the coupling operators $C_{P}$ and $C_{J}$. Second, by casting the problem in a full‐wave FEM setting, complex three‐dimensional geometries, curved interfaces, inhomogeneous materials, anisotropic media, and sheet‐based material models can be treated without reducing the device to a coupled‐mode or equivalent‐circuit description. Third, frequency dispersion is incorporated directly at every harmonic through $L\left(\omega_{n}\right)$ and the frequency‐dependent source‐injection/source‐generation blocks, avoiding slowly varying envelope or weak‐dispersion assumptions commonly invoked in coupled‐mode treatments [27]. In a complementary modal direction, a Floquet‐quasinormal‐mode theory has recently been developed that expands the response of open time‐modulated dispersive scatterers on the quasinormal modes of the static structure, offering efficient spectral insight into mode coupling and Floquet harmonic generation [29].

This full‐wave harmonic‐balance formulation is particularly suitable for dispersive time‐varying photonic structures. Since each harmonic is treated directly in the frequency domain, material dispersion can be evaluated at the actual generated frequency $\omega_{n}$. At the same time, temporal modulation is retained through the coupling operators $C_{P}$ and $C_{J}$, allowing dispersion and fast time modulation to be incorporated simultaneously. The formulation therefore avoids the need for long transient simulations and directly provides the steady‐state amplitudes, phases, and field profiles of all retained Floquet harmonics.

Once the global system is solved, the harmonic‐domain solution can be used to reconstruct the steady‐state time‐domain field, $X\left(r,t\right)=\sum_{n=-N}^{N}X_{n}\left(r\right)e^{j\omega_{n}t}$. Harmonic‐resolved observables are then extracted from the corresponding frequency‐domain fields. The power carried by the nth harmonic through a surface S is computed from the time‐averaged Poynting flux,

(14) $$P_{n}=\frac{1}{2}Re\int_{S}E_{n}\left(r\right)\times H_{n}^{*}\left(r\right)\cdot \hat{n}\;dA,$$

where $H_{n}$ is obtained from Faraday's law as $H_{n}=-{\left[j\omega_{n}\mu \left(\omega_{n}\right)\right]}^{-1}\nabla \times E_{n}$. For port‐based structures, the transmission and reflection coefficients at each harmonic are obtained by projecting the total field onto the corresponding guided modes of the input and output port cross sections. For scattering configurations, harmonic‐resolved scattered power, absorption, and cross sections are computed directly from the near‐field or far‐field quantities at each $\omega_{n}$.

The accuracy of the method is controlled primarily by the harmonic truncation order. Harmonics outside the retained set are neglected, which can introduce truncation error if significant frequency‐converted power is generated beyond the selected range. In practice, convergence is assessed by increasing N and monitoring harmonic‐resolved observables such as $P_{n}$, scattering coefficients, absorption, near‐field distributions, and induced source amplitudes. A typical convergence criterion is

(15) $$\frac{\left|P_{n}^{\left(N+1\right)}-P_{n}^{\left(N\right)}\right|}{\left|P_{n}^{\left(N\right)}\right|}<\eta ,$$

where η is a prescribed tolerance. Once these quantities become insensitive to further expansion of the harmonic basis, the retained Floquet space provides an accurate representation of the steady‐state response.

Overall, the proposed harmonic‐balance framework transforms a dispersive time‐varying electromagnetic problem into a coupled set of full‐wave frequency‐domain subproblems. By representing the time‐varying components through induced secondary polarization and surface‐current sources, the method provides a physically transparent and computationally efficient route for analyzing multi‐harmonic scattering, absorption, and field conversion in time‐modulated photonic structures.

## 3 Results and Discussion

### 3.1 Harmonic‐Resolved Scattering From a Parametrically Pumped Graphene Cylinder

To demonstrate the proposed harmonic‐balance framework in a strongly dispersive and resonant time‐varying surface platform, we consider the scattering response of a dielectric cylinder wrapped by a graphene sheet with a temporally modulated Fermi level. The structure is shown schematically in Figure 2a. The dielectric core has radius a=40μm and relative permittivity $\epsilon_{d}=2.25$, and it is coated by a rolled graphene layer. Such a geometry is not merely a conceptual model: recent experimental work has demonstrated graphene rolls with controllable geometry and chirality, indicating that rolled graphene architectures are a feasible route for realizing cylindrical graphene‐based photonic structures [30]. In addition, optical pump–THz probe experiments have shown that the THz conductivity of graphene can be modified on picosecond and subpicosecond time scales through photoexcitation, providing a physical basis for optically driven temporal modulation of graphene in the THz regime [31, 32].

**FIGURE 2:**
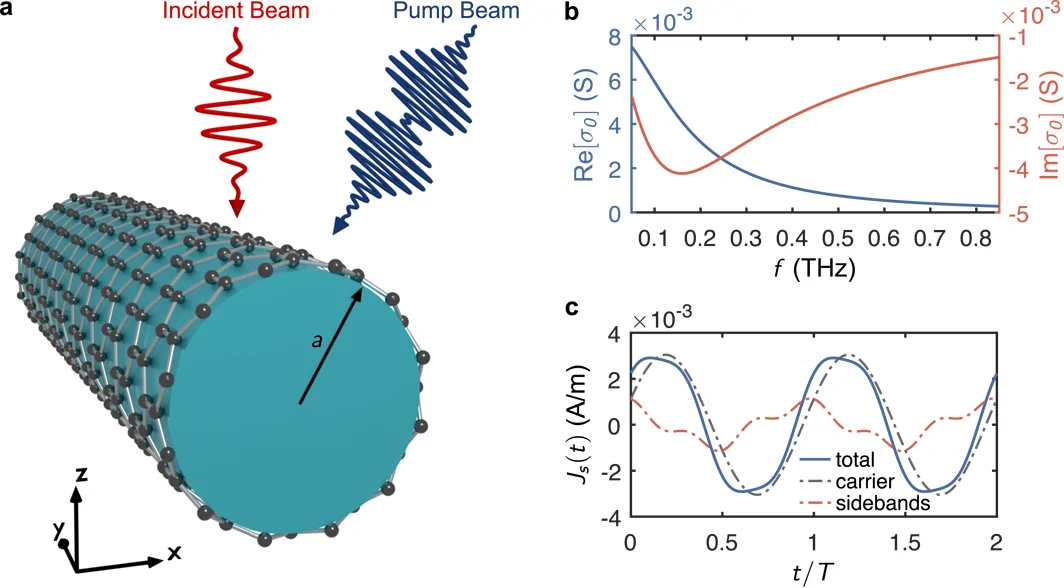
Parametrically pumped rolled graphene cylinder configuration. (a) Schematic of a graphene‐wrapped dielectric cylinder illuminated by an incident beam and driven by an external pump that modulates the graphene Fermi level. The dielectric core has $\epsilon_{r}=2.25$ and radius a=40μm. (b) Real and imaginary parts of the unmodulated graphene surface conductivity $\sigma_{0}$ for $E_{F0}=70\;meV$, showing the dispersive response over the THz band. (c) Steady‐state surface current density $J_{s}\left(t\right)$ induced by a normally incident electric field with $E_{0}=1\;V/m$ and $f_{0}=0.4\;THz$. The total current is decomposed into the carrier contribution and modulation‐induced sidebands with time normalized to the incident‐field period T.

The design starts from the corresponding static, unmodulated graphene cylinder. Under TM illumination, the graphene coating supports localized surface‐plasmon resonances, whose spectral position can be controlled through the cylinder radius, the dielectric permittivity of the core, and the graphene Fermi level. The geometric and material parameters are selected such that, for an unmodulated graphene Fermi level of $E_{F0}=70\;meV$, the static structure exhibits a pronounced localized plasmonic resonance near the operating frequency $f_{0}=0.4\;THz$. The corresponding unmodulated graphene conductivity is shown in Figure 2b where both the real and imaginary parts display the expected dispersive Drude response in the low‐THz range for a graphene relaxation time of τ=1ps. The cylinder is excited by a normally incident TM‐polarized wave with magnetic‐field amplitude $H_{y}=1\;A/m$. After establishing the resonant static design, we introduce temporal modulation by photoexciting the graphene roll. The Fermi level is prescribed as

(16) $$E_{F}\left(t\right)=E_{F0}+\Delta E_{F}\;\cos\left(\Omega t+\alpha \right),$$

where $\Delta E_{F}=40\;meV$. All results are computed with α=π/2; as shown in Section S2 of Supporting Information S1, the harmonic‐resolved cross sections are independent of α, which enters only the phase‐sensitive coherent recombination of the degenerate channel pairs. We intentionally select a fast modulation frequency satisfying $\Omega =2\omega_{0}$, which places the cylinder in the degenerate parametric‐amplification regime. Under this condition, the incident harmonic at $\omega_{0}$ is directly coupled to the down‐converted Floquet harmonic $\omega_{-1}=\omega_{0}-\Omega =-\omega_{0}$. This is the temporal‐scattering analog of modulating a resonant oscillator at twice its natural frequency where the pump supplies energy to the signal and idler channels. In time‐modulated resonant scatterers, this condition is also associated with the opening of a momentum band gap in the equivalent photonic time‐crystal picture, and the amplifying branch inside this gap underlies the parametric enhancement of the scattered fields [33]. Thus, this example is deliberately chosen as a stringent test case: it combines a resonant graphene plasmonic response, strong material dispersion, and rapid temporal modulation at twice the incident frequency.

The time‐domain surface‐current waveform of the modulated graphene sheet is shown in Figure 2c where the graphene is driven by a monochromatic tangential electric field with amplitude 1V/m. The analytical steady‐state form of this time‐domain graphene surface current is derived in Supporting Information S1. We note that the applicability of this constitutive relation to carrier‐density modulation has recently been examined critically, and a corrected relation including a carrier‐removal loss term has been proposed [34]. Such a corrected relation is accommodated by the present framework without structural change, as the removal term merely adds a Toeplitz coupling among the current harmonics (see Section S2 of Supporting Information S1). Even though the excitation is monochromatic, the temporal modulation of the Fermi level generates a multi‐harmonic surface current. The total current can therefore be interpreted as the superposition of the carrier response and modulation‐induced sideband contributions. This behavior is precisely the type of response that the proposed harmonic‐balance formulation is designed to capture: the dispersive graphene surface current is solved simultaneously across the coupled Floquet harmonics rather than by treating the harmonics as independent frequency‐domain problems.

Figure 3 presents the harmonic‐resolved scattering and absorption results. In the static case, shown in Figure 3a, the normalized scattering and absorption cross sections, $\sigma_{\text{sca}}/\left(2a\right)$ and $\sigma_{\text{abs}}/\left(2a\right)$, are confined to the incident harmonic because $\Delta E_{F}=0$. The peak response at $f_{0}=0.4\;THz$ confirms that the selected static parameters place the rolled graphene cylinder near its localized plasmonic resonance. This static resonance provides the electromagnetic enhancement that is subsequently leveraged by the temporal modulation.

**FIGURE 3:**
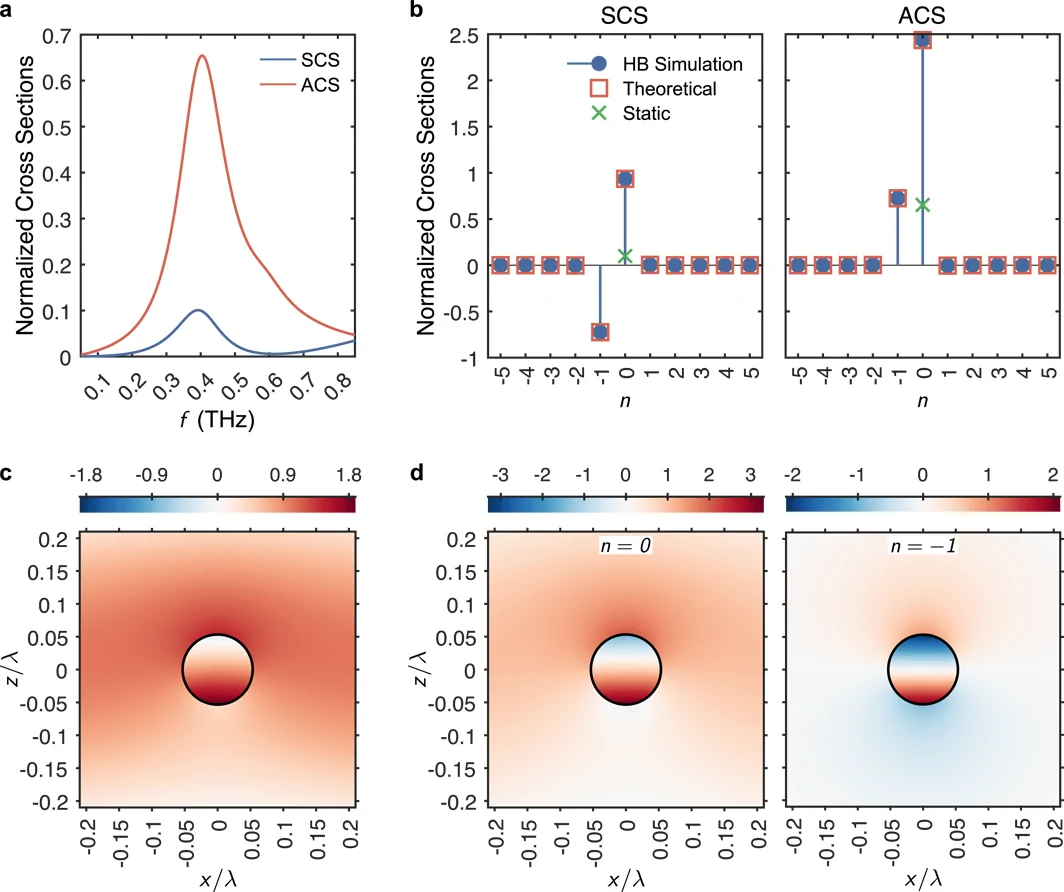
Harmonic‐resolved scattering from a parametrically pumped rolled graphene cylinder. (a) Normalized scattering and absorption cross sections of the static graphene cylinder, obtained in the absence of Fermi‐level modulation, $\Delta E_{F}=0$. (b) Harmonic‐resolved normalized scattering and absorption cross sections of the modulated structure under parametric pumping. The harmonic‐balance FEM results are compared with the analytical solution, whereas the green crosses show the corresponding static response to highlight the redistribution of scattering and absorption among Floquet harmonics. (c) Magnetic‐field distribution, $H_{z}$, at $f_{0}=0.4\;THz$ for the static (unmodulated) graphene cylinder. (d) Magnetic‐field distributions of the zeroth‐order and down‐converted Floquet harmonics, n=0 and n=−1 for the modulated cylinder. Color bars in panels (c, d) are in units of A/m.

When the Fermi level is modulated at $\Omega =2\omega_{0}$, the response changes qualitatively. As shown in Figure 3b, scattering and absorption are redistributed among the Floquet harmonics with the dominant response appearing at the carrier harmonic n=0 and the down‐converted harmonic n=−1. In these simulations, N=5 Floquet harmonics (n=−5,…,5) are retained. The normalized cross sections increase substantially compared with the unmodulated case, indicating that energy supplied by the pump is converted into radiating and absorbed Floquet channels. The harmonic‐balance FEM results agree closely with the analytical Floquet scattering solution derived in Supporting Information S1. This agreement is important because the analytical solution is obtained from the direct cylindrical Floquet formulation, whereas the numerical solution follows from the proposed finite‐element harmonic‐balance framework with time‐varying dispersive surface currents. Their consistency verifies that the framework accurately captures both the graphene dispersion and the modulation‐induced coupling between harmonics.

The field maps in Figure 3c,d provide a complementary view of this process. In the static case, the magnetic‐field distribution $H_{y}$ at $f_{0}=0.4\;THz$ exhibits the localized resonant field pattern associated with the graphene‐coated cylinder. Under parametric pumping, strong field distributions are observed not only at the carrier harmonic n=0, but also at the down‐converted harmonic n=−1. The appearance of a pronounced n=−1 field confirms that the time‐modulated graphene sheet acts as an active frequency‐converting boundary, transferring pump energy into the Floquet sideband. The enhanced near fields and cross sections therefore result from the combined action of the localized graphene plasmon resonance and the parametric coupling induced by the time‐varying Fermi level.

This example highlights the central capability of the harmonic‐balance formulation developed in this work. The method resolves a realistic dispersive time‐varying surface whose modulation frequency is comparable to the signal frequency, whose material response is strongly frequency dependent, and whose scattering behavior is governed by coupled Floquet harmonics. The close agreement with the analytical solution confirms the accuracy of the proposed framework, whereas the parametrically pumped rolled graphene cylinder demonstrates its applicability to resonant time‐varying photonic structures exhibiting harmonic conversion, parametric enhancement, and harmonic‐resolved scattering and absorption.

### 3.2 Optical Space–Time Metasurface Based on Modulated ITO

As a second demonstration, we apply the proposed harmonic‐balance framework to a reflective optical space–time metasurface based on a dispersive time‐modulated epsilon‐near‐zero material. The structure, shown in Figure 4, consists of a binary ${\text{TiO}}_{2}$ grating patterned on top of an ITO layer and backed by a gold reflector. In contrast to the graphene‐cylinder example, where the time‐varying response is introduced through a modulated surface conductivity, here the time‐varying material is volumetric and dispersive. The ITO layer is modeled by a Drude response whose plasma frequency is modulated in time, representing the ultrafast pump‐induced change of the free‐carrier response. This configuration therefore provides a stringent test of the proposed framework because material dispersion and temporal modulation must be treated simultaneously across multiple generated harmonics.

**FIGURE 4:**
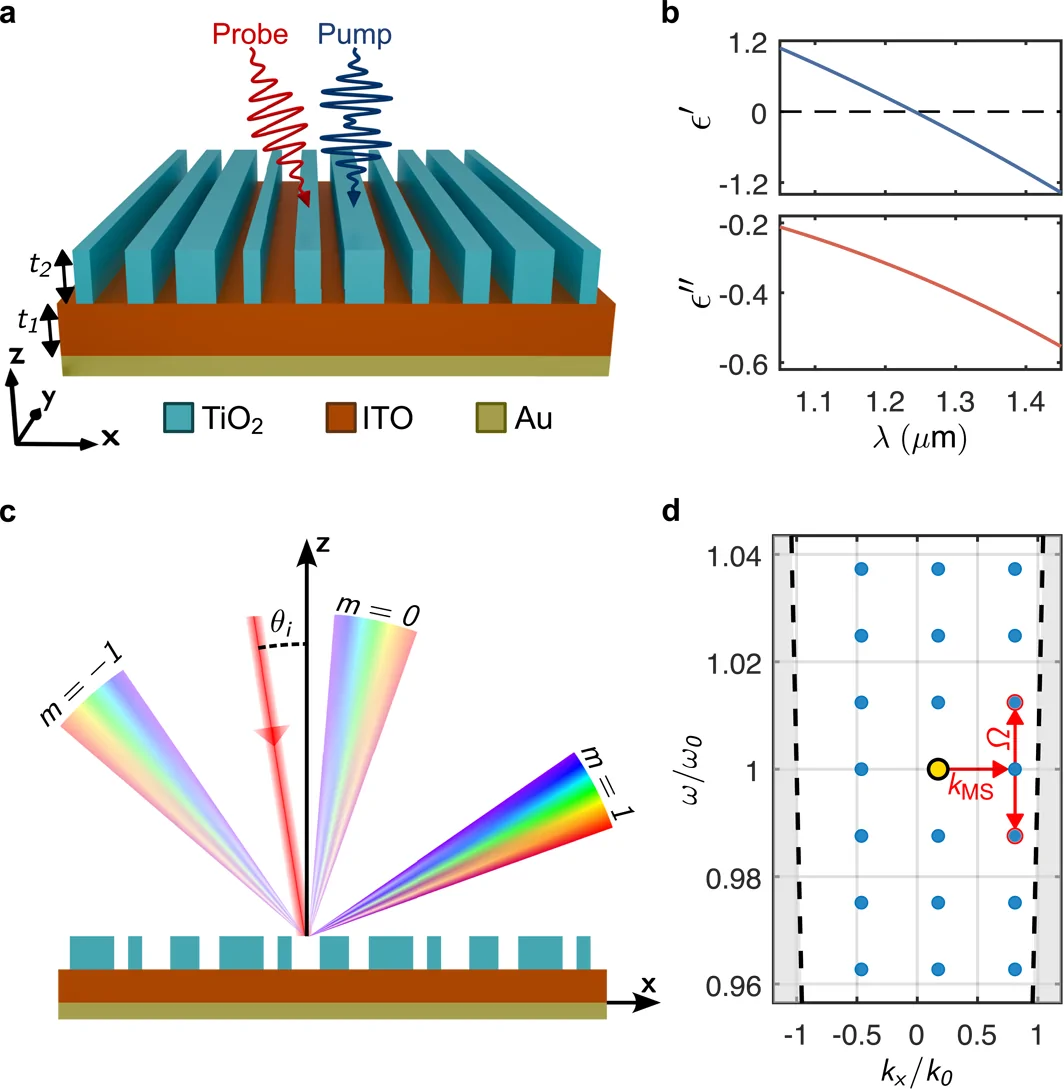
Configuration of an optical space–time metasurface based on modulated ITO. (a) Three‐dimensional schematic of the reflective space–time metasurface composed of a binary corrugated ${\text{TiO}}_{2}$ grating on a time‐varying ITO layer backed by a gold reflector. The probe beam impinges on the metasurface while an external pump modulates the ITO response in time. The ${\text{TiO}}_{2}$ and ITO layer thicknesses are $t_{1}=300\;nm$ and $t_{2}=300\;nm$, respectively. (b) Real and imaginary parts of the ITO permittivity, $\epsilon =\epsilon^{\prime }-j\epsilon^{\prime \prime }$, in the epsilon‐near‐zero spectral region. (c) Two‐dimensional x–z cross section illustrating space–time diffraction from the reflective metasurface, which is uniform along the y direction. The incident beam arrives at $\theta^{\text{inc}}=10{}^{\circ}$, and the diffracted beams correspond to different spatial orders. The bright rainbow beam denotes the targeted space–time diffraction channel, whereas the weaker beams indicate other propagating orders. (d) Dispersion diagram of the space–time diffraction channels. The yellow marker denotes the incident wave at $\omega_{0}$, and the blue markers indicate propagating diffraction orders allowed by the light‐cone constraint. Adjacent orders are separated by $k_{\text{MS}}=2\pi /\Lambda$ along the transverse‐momentum axis and by the modulation frequency Ω along the frequency axis. The red‐circled channels indicate the target orders with spatial index m=+1 for which the metasurface is designed to enhance the diffracted amplitude.

ITO is chosen because transparent conducting oxides exhibit strong optical nonlinearities near their epsilon‐near‐zero wavelength where a small change in carrier response can produce a large change in refractive index and permittivity [35]. In the present design, the unmodulated ITO parameters are selected such that the real part of the permittivity crosses zero near the operating wavelength, $\lambda_{0}=1240\;nm$, as shown in Figure 4b. Operating in this ENZ regime maximizes the effect of plasma‐frequency modulation on the electromagnetic response. This choice is consistent with recent experimental demonstrations of ultrafast wavefront shaping, time‐varying gradient metasurfaces, and space–time optical diffraction based on pumped ITO and related transparent conducting oxides [36, 37, 38].

The dispersive time‐varying ITO response is incorporated through the Drude polarization relation. Denoting $A\left(t\right)=\omega_{p}^{2}\left(t\right)$, the free‐carrier part of the polarization is governed in the frequency domain by a convolution between the plasma‐frequency modulation and the electric field. In compact form, the total polarization is written as

(17) $$P\left(\omega \right)=\epsilon_{0}\left(\epsilon_{\infty }-1\right)E\left(\omega \right)-\frac{\epsilon_{0}}{\omega \left(\omega -j\gamma \right)}\frac{1}{2\pi }\left[A*E\right]\left(\omega \right),$$

where $\epsilon_{\infty }$ is the background permittivity, γ is the damping rate, and ∗ denotes convolution in frequency. For a periodic modulation, this relation reduces to the discrete harmonic coupling equation

(18) $$P_{n}=\epsilon_{0}\left(\epsilon_{\infty }-1\right)E_{n}-\frac{\epsilon_{0}}{\omega_{n}\left(\omega_{n}-j\gamma \right)}\sum_{k}A_{n-k}E_{k},\;\omega_{n}=\omega_{0}+n\Omega .$$

Thus, the Drude denominator is evaluated at each generated harmonic frequency, whereas the Fourier coefficients of A(t) determine the coupling strength between harmonics. This is precisely the situation for which the harmonic‐balance framework is designed: the dispersive material response is not approximated by a static permittivity, and the temporal modulation enters as off‐diagonal coupling in Floquet space.

We assume a single‐tone modulation of the ITO plasma frequency with modulation frequency Ω/2π=3THz. Equivalently, the squared plasma frequency can be written as $A\left(t\right)=A_{0}\left[1+M_{A}\;\cos\left(\Omega t+\alpha \right)\right]$ where $M_{A}=\left(\omega_{p,\max}^{2}-\omega_{p,\min}^{2}\right)/\left(\omega_{p,\max}^{2}+\omega_{p,\min}^{2}\right)$. In the simulations, the pump‐induced swing is chosen such that the pumped and unpumped plasma frequencies differ by 10%, with $\omega_{p,\min}=0.9\;\omega_{p,\max}$. This corresponds to a modulation index of approximately $M_{A}≃0.105$ for the squared plasma frequency. The modulation frequency is chosen close to experimentally accessible multi‐terahertz beat frequencies used in optical space–time modulation; for example, Guo et al. demonstrated a heterodyne‐pump‐induced temporal modulation near 2.8THz in a nonlinear optical metasurface [39].

The purpose of the metasurface is to realize frequency‐converting beam deflection in reflection. Therefore, this device should not be interpreted as a conventional static phase‐gradient metasurface. In a static gradient metasurface, the design target is usually the phase of the reflected field at the incident frequency. Here, instead, the target channel is a space–time diffraction order: the incident wave at $\omega_{0}$ is converted to sideband frequencies $\omega_{n}=\omega_{0}+n\Omega$ and redirected into selected spatial diffraction orders. The diffraction angles are determined by conservation of transverse momentum and energy. For reflected space–time orders, the allowed angles satisfy $\sin\left(\theta_{mn}\right)=\left[\sin\left(\theta_{i}\right)+mK/k_{0}\right]/\left[1+n\Omega /\omega_{0}\right]$, where K=2π/Λ is the supercell momentum, Λ=1950nm is the metasurface period, and $\theta_{i}=10{}^{\circ}$ is the incidence angle. This relation corresponds directly to the dispersion diagram in Figure 4d, where each point represents a space–time diffraction channel. In that diagram, $\theta_{mn}$ can be obtained from the ratio of the transverse‐momentum coordinate to the frequency coordinate equivalently from the slope of the line connecting the origin to the point associated with the corresponding diffraction order. The propagating channels lie above the light lines, whereas the gray shaded region below the light lines corresponds to forbidden, evanescent orders. This interpretation follows the generalized space–time grating theory in which each spatial diffraction order is accompanied by a ladder of temporal harmonics [40].

The design procedure is summarized in Figure 5. The supercell consists of three unit cells, each of length 650nm, so that the total period is Λ=1950nm. Each individual unit cell is subwavelength and therefore supports only the zeroth propagating spatial order, m=0, for the local unit‐cell calculation. We sweep the dielectric filling factor of the ${\text{TiO}}_{2}$ grating and extract the complex reflection coefficient $R_{0,1}$ associated with conversion to the upper sideband, n=1, while remaining in the zeroth spatial order. The amplitude and phase of $R_{0,1}$ are shown in Figure 5a. Because the final device is intended to deflect the frequency‐converted field, the phase library is constructed at the sideband frequency rather than at the incident frequency. Three unit‐cell geometries are then selected such that their $R_{0,1}$ phases are separated by approximately 120°, providing a three‐level discretization of the desired phase progression across the supercell.

**FIGURE 5:**
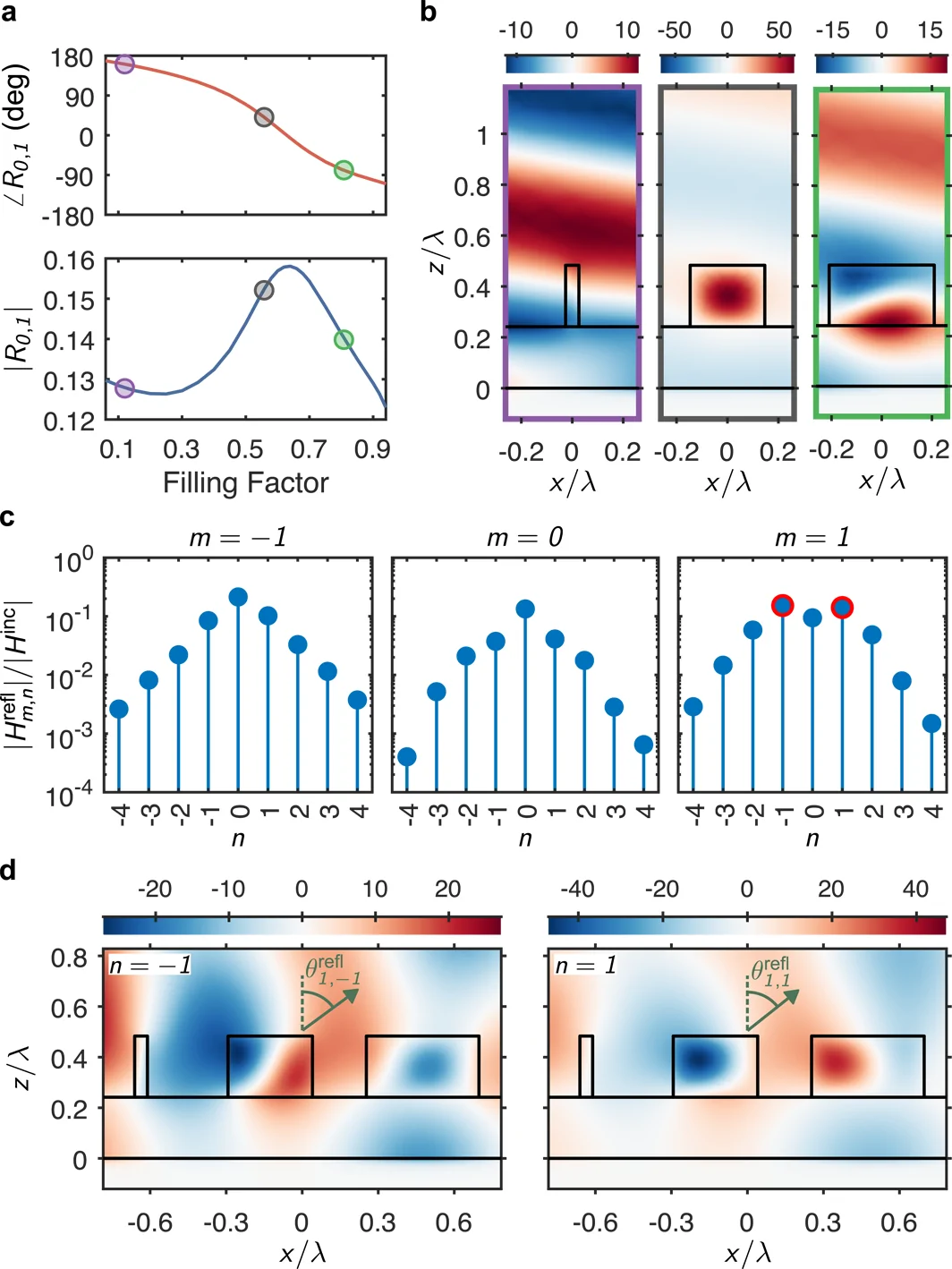
Unit cell selection and harmonic‐resolved response of the optimized ITO space–time metasurface. (a) Phase and amplitude of the unit cell reflection coefficient $R_{0,1}$ for the upper‐side harmonic, n=1, diffracted into the zeroth spatial order, m=0, as a function of dielectric filling factor under TM incidence at $\theta^{\text{inc}}=10{}^{\circ}$. The colored markers denote the selected unit cells. (b) Magnetic‐field distributions, $H_{z}$, for the three selected unit cells in panel (a) with frame colors matching the corresponding markers. (c) Normalized reflected‐field amplitudes of the optimized supercell for spatial orders m=−1,0,1 and harmonics n=−4,…,4 plotted on a common logarithmic scale. The red‐circled points indicate the targeted m=1 space–time diffraction channels. (d) Magnetic‐field profiles of the optimized supercell at $\omega_{-1}$ and $\omega_{1}$, showing dominant reflection into the m=1 order with $\theta_{1,-1}^{\text{refl}}=54.9{}^{\circ}$ and $\theta_{1,1}^{\text{refl}}=53.4{}^{\circ}$, respectively. The excitation power is 1W/m in panels (b, d), and all field maps are in A/m.

The selected unit cells are shown in Figure 5b through their magnetic‐field distributions. These unit cells provide the initial supercell design, but the final metasurface response is further improved by shape optimization of the ${\text{TiO}}_{2}$ grating region. In this optimization step, the positions and widths of the dielectric blocks are adjusted while the time‐varying ITO layer and gold reflector remain fixed. This refinement is important because the optimized device is not merely a local phase‐gradient assembly: near‐field coupling between neighboring grating blocks, the dispersive ENZ response of ITO, the gold‐backed reflective cavity, and the inter‐harmonic coupling all contribute to the final scattering amplitudes. In Supporting Information S1, we further show the normalized amplitudes of the deflected m=1 sideband beams, n=−1 and n=1, as functions of the optimization iteration; see Figure S3a in Supporting Information S1. These curves demonstrate how the targeted sideband amplitudes increase through the gradient‐based optimization. Because the ITO modulation is harmonic, the two first‐order sidebands are generated by the same sinusoidal perturbation of the Drude response. More specifically, for a real single‐tone modulation, the coefficients $A_{+1}$ and $A_{-1}$ are complex conjugates. Consequently, the optimization trajectories of the m=1,n=−1 and m=1,n=1 diffraction amplitudes are strongly correlated, which allows both sideband channels to be enhanced simultaneously without introducing a competing design objective.

Figure 5c shows the harmonic‐resolved reflected‐field amplitudes of the optimized supercell for spatial orders m=−1,0,1 and temporal harmonics n=−4,…,4 corresponding to a harmonic truncation order of N=4. The amplitudes are plotted on a common logarithmic scale, revealing how the incident power is redistributed among multiple space–time diffraction channels. The red‐circled points in Figure 5c correspond to the red‐circled target channels in the dispersion diagram of Figure 4d. At both sideband frequencies $\omega_{-1}$ and $\omega_{1}$, the reflected amplitude in the m=1 spatial diffraction order is larger than the amplitudes of the other spatial orders. Thus, the sideband fields are effectively redirected into the m=1 diffraction channel. This is the central frequency‐converting beam‐deflection mechanism of the metasurface: the incident field at $\omega_{0}$ is converted to sideband harmonics and, at those sideband frequencies, preferentially diffracted into the first spatial order.

For the chosen parameters, the generalized grating relation predicts reflection angles of approximately $\theta_{1,-1}^{\text{refl}}=54.9{}^{\circ}$ and $\theta_{1,1}^{\text{refl}}=53.4{}^{\circ}$ in agreement with the field profiles shown in Figure 5d. These two angles are close because the modulation frequency is much smaller than the optical carrier frequency: $\Omega /\omega_{0}\approx 0.012$ for $\lambda_{0}=1240\;nm$ and Ω/2π=3THz. The same point is evident in the dispersion diagram of Figure 4d where the vertical axis is normalized as $\omega /\omega_{0}$; adjacent temporal harmonics are separated by only a small vertical distance relative to the optical carrier frequency. As a result, the m=1 channels at $\omega_{-1}$ and $\omega_{1}$ have very similar transverse‐momentum‐to‐frequency ratios and therefore very similar reflection angles.

This example demonstrates the ability of the harmonic‐balance framework to analyze and design optical time‐varying metasurfaces containing dispersive volumetric media. Around the ENZ frequency, the material response is inherently dispersive because the real part of the permittivity crosses zero with a large spectral slope, and the wave impedance can vary strongly with frequency. Therefore, modulation of the plasma frequency in a Drude‐type ENZ medium represents modulation of a dispersive material response rather than a simple frequency‐independent perturbation of the permittivity. By solving all retained Floquet harmonics simultaneously, the proposed framework naturally captures the combined effects of ENZ dispersion, time‐dependent Drude coupling, and spatial diffraction from the patterned grating. The resulting design illustrates how dispersive temporal modulation and spatial structuring can be combined to realize frequency‐converting beam steering in a realistic optical metasurface platform.

## 4 Conclusion

We have developed a full‐wave harmonic‐balance framework for dispersive time‐varying photonic structures. The formulation recasts the steady‐state response of a time‐periodic electromagnetic system as a coupled set of frequency‐domain full‐wave problems in Floquet space. By representing the time‐varying components through induced secondary sources, namely volumetric polarization densities and surface current densities, the method provides a unified treatment of modulated bulk media and modulated material sheets. In this representation, material dispersion is evaluated at the physical frequency of each generated harmonic, whereas temporal modulation appears as off‐diagonal coupling between harmonics. The resulting framework therefore captures the combined effects of frequency dispersion, harmonic conversion, parametric energy exchange, and full‐wave spatial scattering without relying on time marching, quasi‐static or weak dispersion approximations.

The validity and versatility of the framework were demonstrated using two representative dispersive time‐varying platforms. In the first example, a parametrically pumped rolled graphene cylinder was analyzed in a regime where the Fermi‐level modulation couples the carrier to the down‐converted Floquet harmonic, and the harmonic‐balance results agreed closely with an analytical Floquet scattering solution, confirming that the method accurately resolves the dispersive graphene surface current, the modulation‐induced harmonic coupling, and the resulting enhancement of scattering and absorption. In the second example, the framework was applied to an optical space–time metasurface consisting of a patterned ${\text{TiO}}_{2}$ grating on a time‐modulated ITO ENZ layer where incorporating the dispersive Drude response of ITO directly in the coupled harmonic system enabled the design and optimization of a reflective frequency‐converting metasurface that redirects the generated sidebands into a selected spatial diffraction order. These examples show that the proposed harmonic‐balance framework can serve not only as an analysis tool, but also as a design platform for active and time‐varying photonic devices with realistic material dispersion and complex geometry, as it directly provides harmonic‐resolved fields, powers, absorption, and diffraction amplitudes, offering a transparent route for quantifying how modulation redistributes electromagnetic energy among frequency‐shifted channels. This capability is essential for emerging space–time photonic systems based on graphene, transparent conducting oxides, ENZ media, resonant metasurfaces, and other strongly dispersive platforms, and the framework therefore provides a general foundation for the full‐wave modeling and inverse design of dispersive time‐varying structures for frequency conversion, parametric amplification, harmonic‐domain beam steering, and dynamic wavefront control.

## Author Contributions

**Mohammad R. Tavakol:** conceptualization, investigation, writing – original draft, methodology, validation, visualization, writing – review and editing, software, formal analysis, data curation. **Wenshan Cai:** conceptualization, funding acquisition, writing – review and editing, project administration, supervision, resources.

## Supporting information


Supporting Information S1

## Data Availability

The data that support the findings of this study are available from the corresponding author upon reasonable request.
