# Supplementary material for: Full‐Wave Harmonic Balance Framework for Dispersive Time‐Varying Photonic Structures
Source: Nanophotonics. 2026 Nov 13;15(22):e70361. doi: 10.1002/nap2.70361 (PMC13768864; doi:10.1002/nap2.70361)
Supplement: Supplementary file 1 [file NAP2-15-e70361-s001.pdf]

Supporting Information

Full-Wave Harmonic Balance Framework for Dispersive Time-Varying Photonic Structures

Mohammad R. Tavakol[1] and Wenshan Cai[1,2,*]

[1]School of Electrical and Computer Engineering, Georgia Institute of Technology, Atlanta, Georgia 30332, United States
[2]School of Materials Science and Engineering, Georgia Institute of Technology, Atlanta, Georgia 30332, United States
[*]Correspondence: wcai@gatech.edu

Contents

1 Surface current response of Fermi-level-modulated graphene 2

2 Floquet scattering from a graphene cylinder with modulated Fermi level 4

3 Complementary results for the pumped rolled graphene cylinder 12

4 Non-sinusoidal Fermi-level modulation of the rolled graphene cylinder 13

5 Floquet scattering from a time-modulated dispersive ITO slab 15

6 Shape Optimization of the ITO-Based Space–Time Metasurface 20

7 Three-dimensional space–time metasurface with subwavelength TiO_2 disks on modulated ITO 23

1 Surface current response of Fermi-level-modulated graphene

Starting from the time-domain constitutive relation for the rolled graphene sheet, whose dispersive response can be expressed via the Drude equation as [S1]

$$\frac{dJ_s(t)}{dt} + \gamma J_s(t) = W(t)E_\phi(t), \tag{S1}$$

we now derive the explicit time-domain surface-current response when the tangential electric field is monochromatic,

$$E_\phi(t) = E_0 \cos(\omega_0 t). \tag{S2}$$

Here, $J_s(t)$ is the azimuthal surface-current density, $\gamma = 1/\tau$ is the carrier relaxation rate, and $W(t)$ is the time-dependent Drude weight of graphene.

For a single-tone modulation of the graphene Fermi level, the Drude weight is written as

$$W(t) = W_0 + \Delta W \cos(\Omega t + \alpha), \tag{S3}$$

where

$$W_0 = \frac{e^2}{\pi\hbar^2} E_{F0}, \qquad \Delta W = \frac{e^2}{\pi\hbar^2} \Delta E_F. \tag{S4}$$

Substituting (S2) and (S3) into the right-hand side of (S1) gives

$$W(t)E_\phi(t) = \left[W_0 + \Delta W \cos(\Omega t + \alpha)\right] E_0 \cos(\omega_0 t). \tag{S5}$$

Using the trigonometric identity

$$\cos A \cos B = \frac{1}{2}\left[\cos(A+B) + \cos(A-B)\right], \tag{S6}$$

we obtain

$$W(t)E_\phi(t) = W_0 E_0 \cos(\omega_0 t) + \frac{\Delta W E_0}{2} \cos[(\omega_0 + \Omega)t + \alpha] + \frac{\Delta W E_0}{2} \cos[(\omega_0 - \Omega)t - \alpha]. \tag{S7}$$

Therefore, the modulation of the Drude weight generates two additional driving frequencies at $\omega_0 + \Omega$ and $\omega_0 - \Omega$, in addition to the original frequency ω_0.

To solve (S1), we use the Fourier transform convention

$$\mathcal{F}\{f(t)\} = f(\omega) = \int_{-\infty}^{\infty} f(t)e^{-j\omega t}\, dt, \qquad \mathcal{F}^{-1}\{f(\omega)\} = \frac{1}{2\pi}\int_{-\infty}^{\infty} f(\omega)e^{j\omega t}\, d\omega. \tag{S8}$$

Using the derivative property

$$\mathcal{F}\left\{\frac{dJ_s(t)}{dt}\right\} = j\omega J_s(\omega), \tag{S9}$$

the Fourier transform of (S1) becomes

$$(\gamma + j\omega)\, J_s(\omega) = \mathcal{F}\{W(t)E_\phi(t)\}. \tag{S10}$$

Hence,

$$J_s(\omega) = \frac{\mathcal{F}\{W(t)E_\phi(t)\}}{\gamma + j\omega}. \tag{S11}$$

The Fourier transform of a shifted cosine is

$$\mathcal{F}\left\{A\cos(\omega_c t + \theta)\right\} = \pi A\left[e^{j\theta}\delta(\omega - \omega_c) + e^{-j\theta}\delta(\omega + \omega_c)\right]. \tag{S12}$$

Applying (S12) to (S7), we obtain

$$\begin{aligned} J_s(\omega) = {} & \frac{\pi W_0 E_0}{\gamma + j\omega}\left[\delta(\omega - \omega_0) + \delta(\omega + \omega_0)\right] \\ & + \frac{\pi \Delta W E_0}{2}\frac{1}{\gamma + j\omega}\left[e^{j\alpha}\delta(\omega - \omega_0 - \Omega) + e^{-j\alpha}\delta(\omega + \omega_0 + \Omega)\right] \\ & + \frac{\pi \Delta W E_0}{2}\frac{1}{\gamma + j\omega}\left[e^{-j\alpha}\delta(\omega - \omega_0 + \Omega) + e^{j\alpha}\delta(\omega + \omega_0 - \Omega)\right]. \end{aligned} \tag{S13}$$

Using the sifting property of the delta function,

$$f(\omega)\delta(\omega - \omega_c) = f(\omega_c)\delta(\omega - \omega_c), \tag{S14}$$

the inverse Fourier transform gives the steady-state current response

$$\begin{aligned} J_s^{\text{ss}}(t) = {} & \frac{W_0 E_0}{2}\left[\frac{e^{j\omega_0 t}}{\gamma + j\omega_0} + \frac{e^{-j\omega_0 t}}{\gamma - j\omega_0}\right] \\ & + \frac{\Delta W E_0}{4}\left[\frac{e^{j[(\omega_0 + \Omega)t + \alpha]}}{\gamma + j(\omega_0 + \Omega)} + \frac{e^{-j[(\omega_0 + \Omega)t + \alpha]}}{\gamma - j(\omega_0 + \Omega)}\right] \\ & + \frac{\Delta W E_0}{4}\left[\frac{e^{j[(\omega_0 - \Omega)t - \alpha]}}{\gamma + j(\omega_0 - \Omega)} + \frac{e^{-j[(\omega_0 - \Omega)t - \alpha]}}{\gamma - j(\omega_0 - \Omega)}\right]. \end{aligned} \tag{S15}$$

Equation (S15) is real-valued because each positive-frequency term is accompanied by its complex-conjugate negative-frequency term. It can therefore be written in the compact real form

$$\begin{aligned} J_s^{\text{ss}}(t) = {} & \frac{W_0 E_0}{\sqrt{\gamma^2 + \omega_0^2}}\cos\left[\omega_0 t - \tan^{-1}\left(\frac{\omega_0}{\gamma}\right)\right] \\ & + \frac{\Delta W E_0}{2\sqrt{\gamma^2 + (\omega_0 + \Omega)^2}}\cos\left[(\omega_0 + \Omega)t + \alpha - \tan^{-1}\left(\frac{\omega_0 + \Omega}{\gamma}\right)\right] \\ & + \frac{\Delta W E_0}{2\sqrt{\gamma^2 + (\omega_0 - \Omega)^2}}\cos\left[(\omega_0 - \Omega)t - \alpha - \tan^{-1}\left(\frac{\omega_0 - \Omega}{\gamma}\right)\right]. \end{aligned} \tag{S16}$$

The complete time-domain solution also includes the homogeneous response of (S1),

$$J_s^{\text{hom}}(t) = Ce^{-\gamma t}, \tag{S17}$$

where C is determined by the initial condition. Thus,

$$J_s(t) = J_s^{\text{ss}}(t) + Ce^{-\gamma t}. \tag{S18}$$

For the steady-state response, or after a time much longer than the relaxation time $\tau = 1/\gamma$, the transient term vanishes and the current is given by (S16).

This result shows explicitly that a monochromatic field at ω_0 produces surface-current harmonics at

$$\omega_0, \qquad \omega_0 + \Omega, \qquad \omega_0 - \Omega. \tag{S19}$$

The sideband currents originate from the temporal modulation of the graphene Drude weight, which mixes the incident electric-field frequency with the modulation frequency.

2 Floquet scattering from a graphene cylinder with modulated Fermi level

We consider two-dimensional TM_y scattering from an infinite dielectric cylinder of radius a, invariant along the y-direction ($\partial/\partial y = 0$). Region I ($r > a$) is free space with permittivity $\varepsilon_{\mathrm{I}} = \varepsilon_0$, and Region II ($r < a$) is a homogeneous dielectric with permittivity $\varepsilon_{\mathrm{II}} = \varepsilon_1$. All media are non-magnetic, with permeability μ_0. The cylindrical coordinates (r, ϕ) are defined in the x–z plane as $x = r\cos\phi$, $z = r\sin\phi$, where $\phi = 0$ corresponds to the $+x$ direction and $\phi = \pi/2$ to the $+z$ direction. Throughout, we adopt the electrical-engineering time convention $e^{j\omega t}$. A conductive coating is modeled as a zero-thickness sheet at $r = a$ with a time-varying surface response that is uniform in ϕ.

Time-domain constitutive relation for graphene. Rather than introducing a conductivity as a function of frequency and time simultaneously, we describe the graphene sheet via a time-domain constitutive relation that links the surface current density to the tangential electric field. In the intraband (Drude-like) regime [S2, S3], the graphene surface current density $J_s(t)$ (tangential to the sheet) satisfies the first-order differential equation

$$\frac{dJ_s(t)}{dt} + \frac{1}{\tau} J_s(t) = W(t)\, E_t(t), \tag{S20}$$

where τ is the carrier relaxation time, $E_t(t)$ is the tangential electric field at the sheet (here $E_t \equiv E_\phi(r = a, \phi, t)$ for the present cylindrical geometry), and $W(t)$ is the (possibly time-dependent) Drude weight.

The Drude weight is related to the graphene Fermi level $E_F(t)$ through

$$W(t) \triangleq \frac{e^2}{\pi\hbar^2} E_F(t). \tag{S21}$$

We note that, for carrier-density modulation, the applicability of (S20) with a time-dependent Drude weight has recently been examined critically [S4]: a decrease of the carrier density removes the current carried by the departing carriers, which adds a removal term $-\frac{1}{2}\mu_r(t)J_s(t)$ to the right-hand side of (S20), with $\mu_r = -N^{-1}dN/dt$ during the decreasing segments. Such a corrected relation is accommodated by the present formulation without structural change: the periodic coefficient $\mu_r(t)$ enters through its Fourier coefficients as an additional Toeplitz matrix acting on the current harmonics, so that the Floquet coupling operator (S53) becomes $\underline{\underline{\sigma_s}} = (\underline{\underline{D}} + \underline{\underline{M_\mu}})^{-1}\underline{\underline{W}}$.

Temporal modulation of the Fermi level. We assume that the Fermi level is harmonically modulated in time as

$$E_F(t) = E_{F0} + \Delta E_F \cos(\Omega t + \alpha), \tag{S22}$$

where Ω is the modulation angular frequency and α is the modulation phase. Consequently, the Drude weight becomes periodic and admits a Fourier-series representation

$$W(t) = \sum_{m\in\mathbb{Z}} W_m e^{jm\Omega t}. \tag{S23}$$

For the single-tone modulation (S22), only $m = 0, \pm 1$ are nonzero and one can write

$$W(t) = W_0 + W_{+1} e^{j\Omega t} + W_{-1} e^{-j\Omega t}, \tag{S24}$$

with Fourier coefficients

$$W_0 = \frac{e^2}{\pi\hbar^2} E_{F0}, \qquad W_{\pm 1} = \frac{e^2}{\pi\hbar^2}\frac{\Delta E_F}{2} e^{\pm j\alpha}. \tag{S25}$$

Floquet harmonics and wavenumbers. Under time-periodic excitation at modulation frequency Ω, the fields develop Floquet harmonics at

$$\omega_n = \omega + n\Omega, \qquad n \in \mathbb{Z}. \tag{S26}$$

We truncate the Floquet index to $n = -N, \ldots, N$, and the azimuthal order to $m = -M, \ldots, M$. The wavenumbers in the two regions for each Floquet harmonic are

$$k_{\mathrm{I},n} = \omega_n \sqrt{\mu_0 \varepsilon_0}, \qquad k_{\mathrm{II},n} = \omega_n \sqrt{\mu_0 \varepsilon_1}. \tag{S27}$$

For TM$_y$ polarization, $\mathbf{H} = \hat{\mathbf{y}} H_y$, and at each Floquet frequency ω_n the tangential electric field satisfies

$$E^{\mathrm{I,II}}_{\phi,n}(r,\phi) = \frac{1}{j\omega_n \varepsilon_{\mathrm{I,II}}} \frac{\partial H^{\mathrm{I,II}}_{y,n}(r,\phi)}{\partial r}. \tag{S28}$$

The incident field is chosen as a plane wave propagating toward $-z$,

$$H^{\mathrm{inc}}_y(r,\phi,t) = H_0\, e^{+jk_{\mathrm{I},0} z}\, e^{j\omega t} = H_0\, e^{+jk_{\mathrm{I},0} r \sin\phi}\, e^{j\omega t}, \tag{S29}$$

which admits the angular expansion [S5]

$$e^{jkr\sin\phi} = \sum_{m=-\infty}^{\infty} J_m(kr) e^{jm\phi}. \tag{S30}$$

Hence, the incident field contains all azimuthal orders m but only the carrier harmonic $n = 0$. Because the geometry is circular and the sheet is uniform in ϕ, distinct azimuthal orders remain decoupled; the plane wave simply provides a known forcing in each m-channel.

We expand the fields as sums of azimuthal harmonics. In Region I ($r > a$), we represent outgoing radiation with Hankel functions of the second kind $H^{(2)}_m$, consistent with the $e^{j\omega t}$ convention. The Floquet-domain magnetic field in Region I is written as

$$H^{\mathrm{I}}_{y,n}(r,\phi) = \sum_{m=-M}^{M} \Big[\delta_{n0} H_0\, J_m(k_{\mathrm{I},n} r) + H_0\, c_{m,n}\, H^{(2)}_m(k_{\mathrm{I},n} r)\Big] e^{jm\phi}. \tag{S31}$$

The coefficients $c_{m,n}$ are unknown and represent the amplitudes of the scattered Floquet harmonics. In Region II ($r < a$), regularity at the origin gives

$$H_{y,n}^{\mathrm{II}}(r,\phi) = \sum_{m=-M}^{M} H_0\, d_{m,n}\, J_m(k_{\mathrm{II},n} r)\, e^{jm\phi}, \tag{S32}$$

where $d_{m,n}$ are internal field coefficients.

Surface current harmonics from the graphene constitutive law. At the sheet $r = a$, the tangential electric field is $E_t(t) \equiv E_\phi(a,\phi,t)$ and the sheet current density is $J_s(t) \equiv J_s(\phi,t)$. Because the Drude weight is periodic, we expand the tangential electric field and sheet current into Floquet harmonics,

$$E_t(t) = \sum_{n\in\mathbb{Z}} E_n(\phi)\, e^{j(\omega+n\Omega)t}, \tag{S33}$$

$$J_s(t) = \sum_{n\in\mathbb{Z}} J_{s,n}(\phi)\, e^{j(\omega+n\Omega)t}, \tag{S34}$$

and likewise expand $W(t)$ as in (S23). Substituting these series into the time-domain constitutive relation (S20) and equating equal time-harmonic terms yields the discrete Floquet relation

$$\left[j(\omega+n\Omega) + \frac{1}{\tau}\right] J_{s,n}(\phi) = \sum_{m\in\mathbb{Z}} W_m\, E_{n-m}(\phi). \tag{S35}$$

For single-tone modulation (S24), only $m = 0, \pm 1$ contribute, and (S35) reduces to the nearest-neighbor coupling law

$$\left[j(\omega+n\Omega) + \frac{1}{\tau}\right] J_{s,n}(\phi) = W_0\, E_n(\phi) + W_{+1}\, E_{n-1}(\phi) + W_{-1}\, E_{n+1}(\phi). \tag{S36}$$

This expression makes explicit that the temporally modulated graphene couples adjacent Floquet harmonics through the coefficients $W_{\pm 1}$, while W_0 produces the usual intraband response in the static limit.

At the sheet $r = a$, the electromagnetic boundary conditions at each Floquet index n are (i) continuity of tangential electric field,

$$E_{\phi,n}^{\mathrm{I}}(a,\phi) = E_{\phi,n}^{\mathrm{II}}(a,\phi), \tag{S37}$$

and (ii) jump of the tangential magnetic field equal to the surface current harmonic,

$$H_{y,n}^{\mathrm{II}}(a,\phi) - H_{y,n}^{\mathrm{I}}(a,\phi) = J_{s,n}(\phi). \tag{S38}$$

Substituting the azimuthal expansions and exploiting the orthogonality of $e^{jm\phi}$, these conditions are enforced independently for each azimuthal order m. The temporal modulation couples different Floquet indices n through (S35)–(S36), but does not mix different m.

To write the resulting equations compactly, we introduce boundary shorthand (evaluated at $r = a$) for each m and n:

$$J_{\mathrm{I},n} = J_m(k_{\mathrm{I},n} a), \quad H_{\mathrm{I},n} = H_m^{(2)}(k_{\mathrm{I},n} a), \quad J_{\mathrm{II},n} = J_m(k_{\mathrm{II},n} a), \tag{S39}$$

and likewise derivatives with respect to the argument,

$$J'_{\mathrm{I},n} = \frac{dJ_m(x)}{dx}\bigg|_{x=k_{\mathrm{I},n}a}, \quad H'_{\mathrm{I},n} = \frac{dH_m^{(2)}(x)}{dx}\bigg|_{x=k_{\mathrm{I},n}a}, \quad J'_{\mathrm{II},n} = \frac{dJ_m(x)}{dx}\bigg|_{x=k_{\mathrm{II},n}a}. \tag{S40}$$

Using $E_{\phi,n}^{\mathrm{I,II}} = -(1/(j\omega_n\varepsilon_{\mathrm{I,II}}))\partial_r H_{y,n}^{\mathrm{I,II}}$, the electric-field continuity condition yields, for each fixed m and each n,

$$\frac{k_{\mathrm{I},n}}{\varepsilon_{\mathrm{I}}}\left(\delta_{n0}J'_{\mathrm{I},n} + c_{m,n}H'_{\mathrm{I},n}\right) = \frac{k_{\mathrm{II},n}}{\varepsilon_{\mathrm{II}}}\, d_{m,n}\, J'_{\mathrm{II},n}. \tag{S41}$$

This relation is diagonal in n and allows the internal coefficient $d_{m,n}$ to be eliminated explicitly:

$$d_{m,n} = \frac{\varepsilon_{\mathrm{II}}k_{\mathrm{I},n}}{\varepsilon_{\mathrm{I}}k_{\mathrm{II},n}}\frac{\delta_{n0}J'_{\mathrm{I},n} + c_{m,n}H'_{\mathrm{I},n}}{J'_{\mathrm{II},n}}. \tag{S42}$$

Closed system for the scattered coefficients. Substituting the eliminated coefficient $d_{m,n}$ into the magnetic jump condition provides a closed set of equations in terms of the unknown scattered coefficients $c_{m,n}$. The coupling among Floquet indices is introduced by the graphene constitutive law (S35) (or (S36) under single-tone modulation), which expresses the surface current harmonics $J_{s,n}(\phi)$ as linear combinations of tangential electric-field harmonics $E_{\phi,n}(a,\phi)$.

For numerical solution and for transparent physical interpretation, it is convenient to write the per-m system as a finite-dimensional matrix equation over the truncated Floquet indices $n = -N, \ldots, N$. We define the unknown coefficient vector

$$\underline{c_m} = \begin{bmatrix} c_{m,-N} & \cdots & c_{m,N} \end{bmatrix}^T, \tag{S43}$$

and the carrier-selection vector

$$\underline{e} = \begin{bmatrix} \delta_{-N,0} & \cdots & \delta_{N,0} \end{bmatrix}^T, \tag{S44}$$

which encodes the fact that the incident field exists only at $n = 0$. We also define diagonal matrices collecting the boundary function values across Floquet harmonics:

$$\underline{\underline{K_{\mathrm{I}}}} = \mathrm{diag}(k_{\mathrm{I},-N}, \ldots, k_{\mathrm{I},N}), \quad \underline{\underline{K_{\mathrm{II}}}} = \mathrm{diag}(k_{\mathrm{II},-N}, \ldots, k_{\mathrm{II},N}), \tag{S45}$$

$$\underline{\underline{J_{\mathrm{I}}}} = \mathrm{diag}(J_{\mathrm{I},-N}, \ldots, J_{\mathrm{I},N}), \quad \underline{\underline{H_{\mathrm{I}}}} = \mathrm{diag}(H_{\mathrm{I},-N}, \ldots, H_{\mathrm{I},N}), \quad \underline{\underline{J_{\mathrm{II}}}} = \mathrm{diag}(J_{\mathrm{II},-N}, \ldots, J_{\mathrm{II},N}), \tag{S46}$$

$$\underline{\underline{J'_{\mathrm{I}}}} = \mathrm{diag}(J'_{\mathrm{I},-N}, \ldots, J'_{\mathrm{I},N}), \quad \underline{\underline{H'_{\mathrm{I}}}} = \mathrm{diag}(H'_{\mathrm{I},-N}, \ldots, H'_{\mathrm{I},N}), \quad \underline{\underline{J'_{\mathrm{II}}}} = \mathrm{diag}(J'_{\mathrm{II},-N}, \ldots, J'_{\mathrm{II},N}), \tag{S47}$$

and the diagonal Floquet-frequency matrix

$$\underline{\underline{\omega}} = \mathrm{diag}(\omega_{-N}, \ldots, \omega_N). \tag{S48}$$

Graphene coupling operator in Floquet space. The graphene constitutive relation (S35) can be written in a compact operator form by defining the diagonal matrix

$$\underline{\underline{D}} = \mathrm{diag}\Big(j\omega_{-N} + \frac{1}{\tau}, \dots, j\omega_N + \frac{1}{\tau}\Big) = j\,\underline{\underline{\omega}} + \frac{1}{\tau}\,\underline{\underline{I}}, \tag{S49}$$

and the Toeplitz matrix $\underline{\underline{W}}$ with elements

$$(\underline{\underline{W}})_{nq} = W_{n-q}, \qquad n, q \in \{-N, \dots, N\}. \tag{S50}$$

Then (S35) becomes, at each ϕ,

$$\underline{\underline{D}}\;\underline{J_s}(\phi) = \underline{\underline{W}}\;\underline{E}(\phi), \qquad \Rightarrow \qquad \underline{J_s}(\phi) = \underline{\underline{D}}^{-1}\underline{\underline{W}}\;\underline{E}(\phi), \tag{S51}$$

where $\underline{E}(\phi) = [E_{-N}(\phi), \dots, E_N(\phi)]^T$ and

$$\underline{J_s}(\phi) = \Big[J_{s,-N}(\phi) \quad \cdots \quad J_{s,N}(\phi)\Big]^T. \tag{S52}$$

For single-tone modulation, $\underline{\underline{W}}$ is tridiagonal since only W_0 and $W_{\pm 1}$ are nonzero.

The time modulation therefore enters through the effective Floquet coupling operator

$$\underline{\underline{\sigma_s}} \triangleq \underline{\underline{D}}^{-1}\,\underline{\underline{W}}, \tag{S53}$$

which maps the tangential electric-field harmonic vector into the surface-current harmonic vector, i.e., $\underline{J_s} = \underline{\underline{\sigma_s}}\,\underline{E}$. Elementwise,

$$(\underline{\underline{\sigma_s}})_{nq} = \frac{W_{n-q}}{j\omega_n + 1/\tau}, \qquad n, q \in \{-N, \dots, N\}. \tag{S54}$$

This expression is consistent with the static limit: when $W(t) = W_0$, one obtains $(\underline{\underline{\sigma_s}})_{nq} = \Big(W_0/(j\omega_n + 1/\tau)\Big)\delta_{nq}$, which recovers the familiar intraband graphene conductivity for each harmonic.

Final matrix system for the scattered coefficients. With the above definition of $\underline{\underline{\sigma_s}}$, the eliminated system for $\underline{c_m}$ can be written as

$$\Big(\underline{\underline{A_m}} + \underline{\underline{\sigma_s}}\;\underline{\underline{C}}\Big)\underline{c_m} = \underline{f_m}, \tag{S55}$$

where $\underline{\underline{A_m}}$ is the effective "static" scattering operator for azimuthal order m, $\underline{\underline{C}}$ maps scattered-field coefficients into the tangential electric field contribution appearing in the surface current, and $\underline{f_m}$ is the forcing vector driven by the incident plane wave. These quantities are given explicitly by

$$\underline{\underline{A_m}} = \frac{\varepsilon_{\mathrm{II}}}{\varepsilon_{\mathrm{I}}}\underline{\underline{K_{\mathrm{I}}K_{\mathrm{II}}}}^{-1}\Big(\underline{\underline{J'_{\mathrm{II}}}}\Big)^{-1}\underline{\underline{J_{\mathrm{II}}}}\,\underline{\underline{H'_{\mathrm{I}}}} - \underline{\underline{H_{\mathrm{I}}}}, \tag{S56}$$

$$\underline{\underline{C}} = \frac{1}{j\varepsilon_{\mathrm{I}}}\,\underline{\underline{K_{\mathrm{I}}}}\,\underline{\underline{\omega}}^{-1}\,\underline{\underline{H'_{\mathrm{I}}}}, \tag{S57}$$

$$\underline{f_m} = \underline{\underline{J_{\mathrm{I}}}}\,\underline{e} - \frac{\varepsilon_{\mathrm{II}}}{\varepsilon_{\mathrm{I}}}\underline{\underline{K_{\mathrm{I}}K_{\mathrm{II}}}}^{-1}\left(\underline{\underline{J'_{\mathrm{II}}}}\right)^{-1}\underline{\underline{J_{\mathrm{II}}}}\,\underline{\underline{J'_{\mathrm{I}}}}\,\underline{e} - \underline{\underline{\sigma_s}}\,\frac{1}{j\varepsilon_{\mathrm{I}}}\underline{\underline{K_{\mathrm{I}}}}\,\underline{\underline{\omega}}^{-1}\,\underline{\underline{J'_{\mathrm{I}}}}\,\underline{e}. \tag{S58}$$

To make the per-element structure fully explicit, let the matrix to be inverted for a given m be denoted as $\underline{\underline{S_m}} = \underline{\underline{A_m}} + \underline{\underline{\sigma_s}}\,\underline{\underline{C}}$. The linear system reads $\sum_{q=-N}^{N}(S_m)_{nq}\,c_{m,q} = (f_m)_n$, with entries

$$(S_m)_{nq} = (A_m)_{nq} + \sum_{p=-N}^{N}(\sigma_s)_{np}\,(C)_{pq}. \tag{S59}$$

Since $\underline{\underline{A_m}}$ and $\underline{\underline{C}}$ are diagonal in the Floquet index, their elements are

$$(A_m)_{nq} = \left[\frac{\varepsilon_{\mathrm{II}}}{\varepsilon_{\mathrm{I}}}\frac{k_{\mathrm{I},n}}{k_{\mathrm{II},n}}\frac{J_{\mathrm{II},n}}{J'_{\mathrm{II},n}}H'_{\mathrm{I},n} - H_{\mathrm{I},n}\right]\delta_{nq}, \tag{S60}$$

$$(C)_{nq} = \left[\frac{1}{j\varepsilon_{\mathrm{I}}}\frac{k_{\mathrm{I},n}}{\omega_n}H'_{\mathrm{I},n}\right]\delta_{nq}. \tag{S61}$$

Combining these facts with (S54) gives the elementwise form

$$(S_m)_{nq} = \left[\frac{\varepsilon_{\mathrm{II}}}{\varepsilon_{\mathrm{I}}}\frac{k_{\mathrm{I},n}}{k_{\mathrm{II},n}}\frac{J_{\mathrm{II},n}}{J'_{\mathrm{II},n}}H'_{\mathrm{I},n} - H_{\mathrm{I},n}\right]\delta_{nq} + \left(\frac{W_{n-q}}{j\omega_n + 1/\tau}\right)\left[\frac{1}{j\varepsilon_{\mathrm{I}}}\frac{k_{\mathrm{I},q}}{\omega_q}H'_{\mathrm{I},q}\right]. \tag{S62}$$

Likewise, the forcing vector elements $(f_m)_n$ can be written explicitly as

$$(f_m)_n = J_{\mathrm{I},n}\,\delta_{n0} - \left[\frac{\varepsilon_{\mathrm{II}}}{\varepsilon_{\mathrm{I}}}\frac{k_{\mathrm{I},n}}{k_{\mathrm{II},n}}\frac{J_{\mathrm{II},n}}{J'_{\mathrm{II},n}}J'_{\mathrm{I},n}\right]\delta_{n0} - \sum_{q=-N}^{N}\left(\frac{W_{n-q}}{j\omega_n + 1/\tau}\right)\left[\frac{1}{j\varepsilon_{\mathrm{I}}}\frac{k_{\mathrm{I},q}}{\omega_q}J'_{\mathrm{I},q}\right]\delta_{q0}. \tag{S63}$$

Since δ_{q0} appears in the last term, the sum collapses to a single contribution, yielding

$$(f_m)_n = J_{\mathrm{I},n}\,\delta_{n0} - \left[\frac{\varepsilon_{\mathrm{II}}}{\varepsilon_{\mathrm{I}}}\frac{k_{\mathrm{I},n}}{k_{\mathrm{II},n}}\frac{J_{\mathrm{II},n}}{J'_{\mathrm{II},n}}J'_{\mathrm{I},n}\right]\delta_{n0} - \left(\frac{W_n}{j\omega_n + 1/\tau}\right)\left[\frac{1}{j\varepsilon_{\mathrm{I}}}\frac{k_{\mathrm{I},0}}{\omega_0}J'_{\mathrm{I},0}\right]. \tag{S64}$$

In practice, solving the problem proceeds as follows. For each azimuthal order $m \in [-M, M]$, one assembles the $(2N+1)\times(2N+1)$ linear system $\underline{\underline{S_m}}\,\underline{c_m} = \underline{f_m}$ using the above elementwise definitions and solves for $\underline{c_m}$. The collection of coefficients $c_{m,n}$ fully determines the scattered field in Region I and is sufficient to compute far-field observables, including the scattering width. If internal fields are required, the eliminated coefficients $d_{m,n}$ are recovered directly from the algebraic relation obtained from tangential electric-field continuity, using the already computed $c_{m,n}$. In the numerical evaluation of the analytical solution, the azimuthal order is truncated to $M = 40$ and the Floquet index to $N = 5$, matching the truncation of the harmonic-balance simulation.

Finally, the scattering width associated with each propagating Floquet harmonic depends only on the scattered coefficients $c_{m,n}$. Under the present normalization, the harmonic-resolved scattering width is

$$\sigma_{\mathrm{sca}}^{(n)} = \frac{4}{k_{\mathrm{I},n}}\sum_{m=-M}^{M}|c_{m,n}|^2, \tag{S65}$$

and the total scattering width is obtained by summing over all the harmonics,

$$\sigma_{\rm sca}^{\rm tot} = \sum_{n=-N}^{N} \sigma_{\rm sca}^{(n)}. \tag{S66}$$

For harmonics with $\omega_n < 0$, the complex amplitudes are the conjugate partners of measurable components at $|\omega_n|$: since the physical field is real, such a channel radiates at the measurable frequency $|\omega_n|$ with the conjugated amplitude, and the outgoing-wave character is preserved under conjugation. The positive-definite power radiated into the n-th Floquet channel, normalized to the incident intensity, is obtained from (S65) with $k_{{\rm I},n}$ replaced by $|k_{{\rm I},n}|$; in the figures, the sign of ω_n is retained in $\sigma_{\rm sca}^{(n)}$ as a bookkeeping label that distinguishes the down-converted negative-frequency channels. Under the degenerate modulation $\Omega = 2\omega_0$, the channel pairs $(n, -n-1)$ share the measurable frequency $(2n+1)\omega_0$ and superpose coherently, so that the field detected at ω_0 is $E_0 + E_{-1}^*$, the signal and the phase-conjugated idler of the degenerate parametric process; the power detected at each measurable frequency then includes a phase-sensitive interference of the paired channels, which is obtained directly from the same complex coefficients.

The modulation phase α enters the Floquet system only through the Toeplitz coefficients $W_{n-q} \propto e^{j(n-q)\alpha}$, from which it follows that $c_{m,n}(\alpha) = e^{jn\alpha} c_{m,n}(0)$, equivalent to a shift of the time origin. All harmonic-resolved cross sections, which depend only on $|c_{m,n}|^2$, are therefore independent of α. The phase sensitivity characteristic of degenerate parametric coupling appears in the coherent recombination of the paired channels: since $c_{m,n}\, c_{-m,-n-1} \propto e^{-j\alpha}$, the interference contribution to the power detected at each measurable frequency oscillates sinusoidally with α. The results in this work are computed with $\alpha = \pi/2$.

This decomposition emphasizes that the time-modulated sheet redistributes scattering among frequency-converted Floquet channels through the Floquet coupling operator $\underline{\underline{\sigma_s}}$, while each azimuthal order m remains an independent scattering channel driven by the plane-wave angular spectrum.

The absorption cross section can be obtained from the time-averaged Joule dissipation on the time-varying graphene sheet. For the n-th Floquet harmonic, the absorbed power per unit length is written as

$$P_{\rm abs}^{(n)} = \frac{1}{2} \int_0^{2\pi} a\, \Re\Big\{ E_{\phi,n}(a,\phi) J_{s,n}^*(a,\phi) \Big\}\, d\phi. \tag{S67}$$

Using the azimuthal expansions of the tangential electric field and the surface current, together with the orthogonality of $e^{jm\phi}$, this becomes

$$P_{\rm abs}^{(n)} = \pi a \sum_{m=-M}^{M} \Re\Big\{ E_{\phi,m,n}(a) J_{s,m,n}^*(a) \Big\}. \tag{S68}$$

Therefore, the harmonic-resolved absorption cross section is

$$\sigma_{\rm abs}^{(n)} = \frac{P_{\rm abs}^{(n)}}{I_0} = \frac{\pi a}{I_0} \sum_{m=-M}^{M} \Re\Big\{ E_{\phi,m,n}(a) J_{s,m,n}^*(a) \Big\}, \tag{S69}$$

where I_0 is the incident intensity at the carrier frequency. The total absorption cross section is obtained by summing over the retained Floquet harmonics,

$$\sigma_{\text{abs}} = \sum_{n=-N}^{N} \sigma_{\text{abs}}^{(n)}. \tag{S70}$$

Unlike the scattering width, the harmonic-resolved absorption cross section is intrinsically signed: a negative value of $\sigma_{\text{abs}}^{(n)}$ indicates that the time-modulated sheet does net work on the field at that harmonic, so that the channel is parametrically emitting, with the energy supplied by the modulation. In the figures, the sign of ω_n is likewise attached to $\sigma_{\text{abs}}^{(n)}$ for consistency with the labeling of the scattering channels.

After solving the harmonic-balance system for the scattered coefficients, the boundary tangential electric-field vector for each azimuthal order is obtained from the same boundary relation used in the matrix formulation. The corresponding surface-current vector follows from the Floquet admittance relation of the graphene sheet. The scalar quantities $E_{\phi,m,n}(a)$ and $J_{s,m,n}(a)$ in (S69) are the n-th entries of these vectors.

For numerical evaluation, the quantities appearing in (S69) are obtained directly from the same matrices used in the harmonic-balance system. For each azimuthal order m, after solving

$$\underline{\underline{S_m}}\,\underline{c_m} = \underline{f_m}, \tag{S71}$$

the boundary tangential electric-field vector is

$$\underline{E_{\phi,m}}(a) = \underline{E_{\phi,m}^{\text{inc}}}(a) + \underline{\underline{C}}\,\underline{c_m}, \tag{S72}$$

where

$$\underline{E_{\phi,m}^{\text{inc}}}(a) = \frac{1}{j\varepsilon_{\text{I}}}\,\underline{\underline{K_{\text{I}}}}\,\underline{\underline{\omega}}^{-1}\,\underline{\underline{J_{\text{I}}'}}\,\underline{e}. \tag{S73}$$

Here, $\underline{\underline{C}}\,\underline{c_m}$ is the scattered-field contribution to the tangential electric field at $r = a$, with

$$\underline{\underline{C}} = \frac{1}{j\varepsilon_{\text{I}}}\,\underline{\underline{K_{\text{I}}}}\,\underline{\underline{\omega}}^{-1}\,\underline{\underline{H_{\text{I}}'}}. \tag{S74}$$

The corresponding surface-current vector is obtained from the Floquet surface-conductivity relation

$$\underline{J_{s,m}} = \underline{\underline{\sigma_s}}\,\underline{E_{\phi,m}}(a). \tag{S75}$$

Therefore,

$$E_{\phi,m,n}(a) = \left[\underline{E_{\phi,m}^{\text{inc}}}(a) + \underline{\underline{C}}\,\underline{c_m}\right]_n, \tag{S76}$$

and

$$J_{s,m,n}(a) = \left[\underline{\underline{\sigma_s}}\left(\underline{E_{\phi,m}^{\text{inc}}}(a) + \underline{\underline{C}}\,\underline{c_m}\right)\right]_n. \tag{S77}$$

Substituting these entries into the harmonic-resolved absorption expression gives

$$\sigma_{\text{abs}}^{(n)} = \frac{\pi a}{I_0} \sum_{m=-M}^{M} \Re\left\{ \left[\underline{E_{\phi,m}^{\text{inc}}}(a) + \underline{\underline{C}}\,\underline{c_m}\right]_n \left[\underline{\underline{\sigma_s}}\left(\underline{E_{\phi,m}^{\text{inc}}}(a) + \underline{\underline{C}}\,\underline{c_m}\right)\right]_n^* \right\}. \tag{S78}$$

3 Complementary results for the pumped rolled graphene cylinder

To further validate the harmonic-balance formulation for the Fermi-level-modulated rolled graphene cylinder, we provide additional harmonic-resolved scattering and absorption results in Fig. S1. In the main text, the dominant harmonics are sufficient to describe the principal frequency-conversion response. However, many higher-order Floquet harmonics have cross sections that are several orders of magnitude smaller than the main response and therefore cannot be clearly distinguished on a conventional linear scale. To make these weak harmonic channels visible, we plot the normalized scattering and absorption cross sections using a μ-law companded scale.

The μ-law compression function is defined as

$$F(x) = \operatorname{sgn}(x) \frac{\log(1 + \mu|x|)}{\log(1 + \mu)},$$

with the corresponding inverse mapping

$$F^{-1}(y) = \operatorname{sgn}(y) \frac{1}{\mu} \left[(1 + \mu)^{|y|} - 1 \right].$$

This nonlinear mapping preserves the sign of the input while compressing a large dynamic range into a finite visual interval. Therefore, very small cross-section values, including those associated with harmonics far from the dominant harmonic channels, become distinguishable without obscuring the stronger response. As shown in Fig. S1, the harmonic-balance results remain in excellent agreement with the analytical Floquet scattering formulation developed in Sec. 2 across the full set of resolved harmonics. In particular, the agreement persists even for weak harmonic responses with normalized cross sections approaching 10^{-10}, demonstrating that the proposed harmonic-balance framework accurately captures both the dominant and the highly suppressed frequency-converted channels.

Figure S2 provides additional field-level insight into the same pumped rolled graphene cylinder configuration. Specifically, Fig. S2(a) compares the far-field electric-field pattern of the static cylinder with the dominant Floquet harmonics of the modulated structure. For the modulated case, only the $n = 0$ and $n = -1$ harmonics are shown because these channels dominate the scattered response. The comparison illustrates how temporal modulation redistributes the scattered field among Floquet harmonics and can enhance the far-field amplitude relative to the corresponding static configuration.

The same modulation-induced enhancement is also evident in the graphene surface current distribution shown in Fig. S2(b). The surface current density is plotted as a function of the polar angle ϕ, again comparing the static case with the $n = 0$ and $n = -1$ harmonics of the pumped structure. These current profiles clarify the physical origin of the far-field response: modulation of the graphene Fermi level modifies the induced surface current on the rolled sheet, and the resulting harmonic surface currents act as sources for the frequency-converted scattered fields. Overall, Fig. S1 serves as a stringent accuracy benchmark for the proposed harmonic-balance framework by comparing it with the analytical Floquet scattering formulation over a large dynamic range. In contrast, Fig. S2 provides complementary

field- and current-level results, showing that the far-field and induced surface current responses are consistent with the scattering and absorption cross section trends reported for the pumped rolled graphene cylinder.

For reference, the harmonic-balance simulations of the pumped cylinder retain $N = 5$ Floquet harmonics and are discretized with quadratic Lagrange elements; the time-varying graphene sheet is meshed with a maximum element size of $0.004\lambda_0$, so that the surface currents parametrically induced in the degenerate regime are accurately resolved, with the element size growing gradually away from the sheet. The fully coupled multiharmonic system, comprising 742,654 degrees of freedom, is factorized and solved by a direct sparse solver in a single stationary step, requiring approximately 13 s for the solve (about 26 s including assembly) and less than 4 GB of memory on a ten-core Intel Xeon workstation.

4 Non-sinusoidal Fermi-level modulation of the rolled graphene cylinder

The results presented thus far assume a single-tone modulation, for which only W_0 and $W_{\pm 1}$ are nonzero and the coupling among Floquet harmonics is restricted to nearest neighbors. The harmonic-balance formulation of the main text, however, places no restriction on the modulation spectrum. An arbitrary periodic waveform enters only through its temporal Fourier coefficients, which populate additional off-diagonal bands of the source-generation operators [Eq. (7) of the main text]. To demonstrate this capability, we revisit the parametrically pumped rolled graphene cylinder of the main text and replace the sinusoidal Fermi-level modulation with triangular and rectangular waveforms. All other parameters, including a, ϵ_d, E_{F0}, ΔE_F, τ, f_0, and $\Omega = 2\omega_0$, are identical to those used in the main text.

Modulation waveforms. We generalize (S22) as

$$E_F(t) = E_{F0} + \Delta E_F \, f(\Omega t + \alpha), \tag{S79}$$

where $f(\theta)$ is a 2π-periodic, zero-mean waveform with unit peak amplitude; the choice $f(\theta) = \cos\theta$ recovers (S22). The triangular and rectangular profiles admit the Fourier-series representations

$$f_{\text{tri}}(\theta) = \frac{8}{\pi^2} \sum_{n \text{ odd}} \frac{\cos(n\theta)}{n^2}, \qquad f_{\text{rect}}(\theta) = \frac{4}{\pi} \sum_{n \text{ odd}} \frac{(-1)^{(n-1)/2} \cos(n\theta)}{n}. \tag{S80}$$

Both waveforms possess half-wave symmetry, $f(\theta + \pi) = -f(\theta)$, so their spectra contain only odd harmonics. Substituting (S79) into (S21) gives the Drude-weight Fourier coefficients

$$W_0 = \frac{e^2}{\pi\hbar^2} E_{F0}, \qquad W_{\pm n} = \frac{e^2}{\pi\hbar^2} \frac{\Delta E_F}{2} \, c_n \, e^{\pm jn\alpha} \quad (n \text{ odd}), \qquad W_{\pm n} = 0 \quad (n \text{ even}, \; n \neq 0), \tag{S81}$$

with $c_n = 8/(\pi^2 n^2)$ for the triangular profile and $c_n = (4/\pi)(-1)^{(n-1)/2}/n$ for the rectangular profile. The sinusoidal case corresponds to $c_1 = 1$ with all other $c_n = 0$.

Harmonic truncation of the modulation. The ideal waveforms contain infinitely many harmonics, so the modulation is truncated to $|n| \leq L$. We retain harmonics up to $L = 3$ for the triangular profile and $L = 5$ for the rectangular profile. The fraction of the modulation power, i.e., the time-averaged variance of $E_F(t)$ about E_{F0}, captured by the truncated series is

$$\eta_L = \frac{\sum_{n \text{ odd}, n \leq L} c_n^2}{\sum_{n \text{ odd}} c_n^2}. \tag{S82}$$

For the triangular profile, $\sum_{n \text{ odd}} n^{-4} = \pi^4/96$, which gives $\eta_3 = (1 + 3^{-4})/(\pi^4/96) \approx 99.8\%$. For the rectangular profile, $\sum_{n \text{ odd}} n^{-2} = \pi^2/8$, which gives $\eta_5 = (1 + 3^{-2} + 5^{-2})/(\pi^2/8) \approx 93.3\%$. The fundamental component alone carries approximately 98.6% and 81.1% of the modulation power, respectively. The slower $1/n$ decay of the rectangular spectrum, associated with the discontinuities of the waveform, is the reason a larger number of harmonics is required. The ideal waveforms and their truncated reconstructions are compared with the sinusoidal profile in Figs. S3(a) and S3(c). The triangular reconstruction is nearly indistinguishable from the ideal waveform, except for a slight rounding at its extrema. The rectangular reconstruction exhibits Gibbs oscillations near the transitions and overshoots the ideal levels. The reconstructed Fermi level nevertheless remains positive throughout the modulation period, so the intraband Drude description of (S20) remains applicable. The truncated waveforms are used as the prescribed modulation in both the harmonic-balance simulation and the analytical solution.

Coupling structure in Floquet space. For these waveforms, the Toeplitz matrix $\underline{\underline{W}}$ of (S50) is no longer tridiagonal. Its nonzero diagonals lie at offsets 0, ± 1, and ± 3 for the triangular profile, with additional diagonals at ± 5 for the rectangular profile. The Floquet coupling operator $\underline{\underline{\sigma_s}}$ of (S53) therefore becomes block-banded with a harmonic bandwidth set by L. In the harmonic-balance framework, this corresponds to additional off-diagonal blocks in the sheet source-generation operator $\mathcal{C}_J$. No other modification of either the analytical formulation or the numerical framework is required: only the Fourier coefficients of the modulation change. For a fixed Floquet truncation order N, the number of unknowns is unchanged, and only the number of nonzero coupling blocks per row increases, from three for sinusoidal modulation to five and seven for the triangular and rectangular profiles, respectively.

Surface-current response under prescribed driving. To isolate the effect of the modulation spectrum, we first consider the graphene sheet driven by a prescribed monochromatic tangential field, as in Sec. 1. Generalizing (S15) and (S16) to an arbitrary periodic Drude weight gives the steady-state current

$$J_s^{\text{ss}}(t) = \Re\left\{\sum_n J_{s,n}\, e^{j\omega_n t}\right\}, \qquad J_{s,n} = \frac{W_n\, E_0}{\gamma + j\omega_n}, \qquad \omega_n = \omega_0 + n\Omega, \tag{S83}$$

which reduces to (S16) for single-tone modulation. Each Fourier component of $W(t)$ generates exactly one current sideband. Under a prescribed driving field, the current spectrum therefore mirrors the modulation spectrum: only the odd-order sidebands $n = \pm 1, \pm 3$ appear for the triangular profile, and $n = \pm 1, \pm 3, \pm 5$ for the rectangular profile, as shown in

Figs. S3(b) and S3(d). Relative to the sinusoidal case, the first-order sideband amplitudes scale with c_1. They are reduced by a factor of $8/\pi^2 \approx 0.81$ for the triangular profile and increased by $4/\pi \approx 1.27$ for the rectangular profile. All sidebands are further weighted by the dispersive Drude factor $1/(\gamma + j\omega_n)$, which is evaluated at the physical frequency of each harmonic. Because $\Omega = 2\omega_0$, for $n \geq 1$ the lower sidebands satisfy $|\omega_{-n}| = (2n-1)\omega_0$, whereas the upper sidebands satisfy $|\omega_n| = (2n+1)\omega_0$. This explains why each lower sideband exceeds its upper counterpart.

Harmonic-resolved scattering and absorption. In the full scattering problem, the tangential field at the sheet is no longer prescribed. It is determined self-consistently, because each current harmonic radiates and modifies the field at the sheet, which in turn drives further current harmonics. Repeated action of the modulation then couples harmonics through combinations of odd modulation orders. For example, the harmonic $n = 2$ can be reached through two successive first-order up-conversion steps, or through a third-order up-conversion step followed by a first-order down-conversion step. Consequently, although the modulation spectrum contains only odd-order components, all Floquet harmonics, including the even-order ones, are populated in the scattering response. This multi-step inter-harmonic coupling is precisely what is captured by solving the global harmonic-balance system rather than treating the harmonics independently.

Figure S4 presents the harmonic-resolved normalized scattering and absorption cross sections for both modulation profiles, plotted on the μ-law companded scale introduced in Sec. 3. The compression parameter is set to $\mu = 10^{15}$ for the triangular profile and $\mu = 10^{10}$ for the rectangular profile to match the dynamic range of each response. For both profiles, the harmonic-balance results agree closely with the analytical Floquet scattering solution of Sec. 2 across the full set of resolved harmonics, including channels that are many orders of magnitude weaker than the dominant response. As in the sinusoidal case, the dominant response remains concentrated in the carrier harmonic $n = 0$ and the down-converted harmonic $n = -1$, because the fundamental modulation component continues to couple ω_0 to $\omega_{-1} = -\omega_0$ in the degenerate parametric regime. The higher-order modulation components, however, broaden the distribution of power among Floquet harmonics, and this broadening is more pronounced for the rectangular profile owing to its slowly decaying modulation spectrum.

These results confirm that the proposed harmonic-balance framework handles non-sinusoidal temporal modulation without any change to its formulation. The modulation waveform enters only through its Fourier coefficients, which determine the bandwidth and strength of the off-diagonal coupling in Floquet space, while material dispersion continues to be evaluated at the physical frequency of each harmonic.

5 Floquet scattering from a time-modulated dispersive ITO slab

To provide an independent benchmark for the volumetric-polarization version of the harmonic-balance framework, we derive here an analytical Floquet solution for a single free-standing

ITO slab whose plasma frequency is modulated in time. The slab has thickness $L = 300$ nm, and its material and modulation parameters are identical to those of the ITO layer used in the space–time metasurface of the main text and in the disk-array metasurface of Sec. 7, but the slab is considered alone, with air on both sides and no grating or reflector, so that the structure is laterally uniform and admits a semi-analytical multiharmonic solution. The derivation follows the modal Floquet formalism developed in [S6] for instantaneous (non-dispersive) time-varying slabs, generalized here to a dispersive Drude medium with modulated plasma frequency.

The geometry is stratified along z: Region I ($z > L$) is air and contains the incident wave, the time-varying (TV) slab occupies $0 < z < L$, and Region III ($z < 0$) is air and contains the transmitted harmonics. We consider TM polarization, $\mathbf{H} = H_y\hat{\mathbf{y}}$, with invariance along y and the time convention $e^{j\omega t}$, so that $e^{-jk_z z}$ propagates toward $+z$ and $e^{+jk_z z}$ toward $-z$. A TM plane wave at angular frequency ω_0 impinges from Region I, propagating toward $-z$ with transverse wavenumber $k_{x,\text{inc}} = k_0 \sin\theta_{\text{inc}}$, which is conserved across all harmonics. The Floquet frequencies are

$$\omega_n = \omega_0 + n\Omega, \qquad n = -N, \dots, N. \tag{S84}$$

As in the main text, the squared plasma frequency of the ITO slab is modulated as $A(t) = \omega_p^2(t) = A_0\,[1 + M_A \cos(\Omega t + \alpha)]$, with Fourier coefficients

$$A_0, \qquad A_{\pm1} = \frac{A_0 M_A}{2}\, e^{\pm j\alpha}, \qquad A_{|\ell|>1} = 0. \tag{S85}$$

Floquet permittivity operator of the dispersive modulated slab

The dispersive time-varying response of ITO is described by the Drude polarization relation of the main text [Eq. (18)],

$$P_n = \epsilon_0(\epsilon_\infty - 1)E_n - \frac{\epsilon_0}{\omega_n(\omega_n - j\gamma)} \sum_q A_{n-q} E_q. \tag{S86}$$

Writing the displacement harmonics as $D_n = \epsilon_0 E_n + P_n = \epsilon_0 \sum_q \left(\underline{\underline{\epsilon_r}}\right)_{nq} E_q$ defines the effective Floquet permittivity operator

$$\left(\underline{\underline{\epsilon_r}}\right)_{nq} = \epsilon_\infty\, \delta_{nq} - \frac{A_{n-q}}{\omega_n(\omega_n - j\gamma)}, \tag{S87}$$

where n is the observation (row) harmonic and q the excitation (column) harmonic. Unlike the Toeplitz permittivity operator of an instantaneous time-varying dielectric, the operator (S87) is *not* Toeplitz: the Drude denominator is evaluated at the row frequency ω_n, so its elements depend on n and q separately rather than only on $n - q$. This is precisely the dispersive feature that the harmonic-balance framework is designed to capture, namely that each generated harmonic experiences the material response at its own physical frequency. The diagonal entries, $\epsilon_\infty - A_0/[\omega_n(\omega_n - j\gamma)]$, reduce to the static Drude permittivity evaluated at each ω_n; for $M_A = 0$ the operator becomes diagonal and all harmonics decouple, while for the single-tone modulation (S85) it is tridiagonal, corresponding to nearest-neighbor coupling in Floquet space.

Eigenmodes of the modulated dispersive slab

Inside the slab, the multiharmonic Maxwell curl equations read

$$\nabla \times \underline{\mathbf{E}} = -j\mu_0 \,\underline{\underline{\omega}}\, \underline{\mathbf{H}}, \qquad \nabla \times \underline{\mathbf{H}} = j\epsilon_0 \,\underline{\underline{\omega}}\, \underline{\underline{\epsilon_r}}\, \underline{\mathbf{E}}, \tag{S88}$$

with $\underline{\underline{\omega}} = \mathrm{diag}(\ldots, \omega_{-1}, \omega_0, \omega_{+1}, \ldots)$. Eliminating $\underline{\mathbf{E}}$ yields the multiharmonic Helmholtz equation for the H_y harmonic vector,

$$\nabla^2 \underline{H} + \frac{1}{c^2}\,\underline{\underline{\omega}}\,\underline{\underline{\epsilon_r}}\,\underline{\underline{\omega}}\,\underline{H} = 0. \tag{S89}$$

Substituting the ansatz $\underline{H}(x,z) = e^{-jk_{x,\mathrm{inc}}x}\, e^{\mp jk_z^{\mathrm{TV}}z}\,\underline{H}$ gives the generalized eigenvalue problem

$$\left[k_{x,\mathrm{inc}}^2\,\underline{\underline{I}} + \left(k_z^{\mathrm{TV}}\right)^2 \underline{\underline{I}} - \frac{1}{c^2}\,\underline{\underline{\omega}}\,\underline{\underline{\epsilon_r}}\,\underline{\underline{\omega}}\right]\underline{H} = 0, \tag{S90}$$

formally identical to the dispersion problem of an instantaneous time-varying slab, but with the dispersive coupling kernel

$$\left(\underline{\underline{\omega}}\,\underline{\underline{\epsilon_r}}\,\underline{\underline{\omega}}\right)_{nq} = \epsilon_\infty\,\omega_n^2\,\delta_{nq} - \frac{A_{n-q}\,\omega_q}{\omega_n - j\gamma}. \tag{S91}$$

Diagonalizing the $(2N{+}1)\times(2N{+}1)$ matrix $\frac{1}{c^2}\underline{\underline{\omega}}\,\underline{\underline{\epsilon_r}}\,\underline{\underline{\omega}} - k_{x,\mathrm{inc}}^2\underline{\underline{I}}$ yields the eigenvalues $\left(k_{z,m}^{\mathrm{TV}}\right)^2$ and eigenvectors $\underline{\Psi}_m^{\mathrm{TV}}$, collected into the modal matrix

$$\underline{\underline{\Psi}}^{\mathrm{TV}} = \left[\ldots, \underline{\Psi}_{-1}^{\mathrm{TV}}, \underline{\Psi}_0^{\mathrm{TV}}, \underline{\Psi}_{+1}^{\mathrm{TV}}, \ldots\right], \tag{S92}$$

whose rows index the temporal harmonics n and whose columns index the eigenmodes m. Each eigenfunction,

$$\psi_m^{\mathrm{TV}}(t) = \sum_n \Psi_{m,n}^{\mathrm{TV}}\, e^{j\omega_n t}, \tag{S93}$$

is a mixture of harmonics dominated by $e^{j\omega_m t}$. The branch of each $k_{z,m}^{\mathrm{TV}}$ is selected with $\Im\{k_{z,m}^{\mathrm{TV}}\} \le 0$, so that $e^{-jk_{z,m}^{\mathrm{TV}}z}$ decays toward $+z$ and $e^{+jk_{z,m}^{\mathrm{TV}}z}$ decays toward $-z$. Since $\underline{\underline{\epsilon_r}}$ is non-Hermitian due to both loss and modulation, no symmetry of the eigenpairs is assumed.

Field expansions and boundary conditions

The magnetic field in the three regions is expanded as

$$H^{\mathrm{I}}(x,z;t) = e^{-jk_{x,\mathrm{inc}}x}\left[e^{+jk_{z,\mathrm{inc}}(z-L)}e^{j\omega_0 t} + \sum_n r_n\, e^{-jk_{z,n}(z-L)}\, e^{j\omega_n t}\right], \qquad z > L, \tag{S94}$$

$$H^{\mathrm{TV}}(x,z;t) = e^{-jk_{x,\mathrm{inc}}x}\sum_m \left(a_m^{\mathrm{TV}} e^{-jk_{z,m}^{\mathrm{TV}}z} + b_m^{\mathrm{TV}} e^{+jk_{z,m}^{\mathrm{TV}}(z-L)}\right)\psi_m^{\mathrm{TV}}(t), \qquad 0 < z < L, \tag{S95}$$

$$H^{\mathrm{III}}(x,z;t) = e^{-jk_{x,\mathrm{inc}}x}\sum_n t_n\, e^{+jk_{z,n}z}\, e^{j\omega_n t}, \qquad z < 0, \tag{S96}$$

where a_m^{TV} and b_m^{TV} are the amplitudes of the $+z$- and $-z$-propagating slab eigenmodes, respectively, $k_{z,n} = \sqrt{(\omega_n/c)^2 - k_{x,\mathrm{inc}}^2}$ with $\Im\{k_{z,n}\} < 0$ for evanescent orders, $k_n = \omega_n/c$,

and $\theta_n = \cos^{-1}(k_{z,n}/k_n)$. Note that the air-region sums run over the harmonics n, which are uncoupled plane waves in the static regions, whereas the slab sum runs over the eigenmodes m, each of which mixes all harmonics through $\psi_m^{\mathrm{TV}}(t)$. The $-z$-going amplitudes are phase-referenced to the entry interface $z = L$ and the $+z$-going amplitudes to the exit interface $z = 0$, so that only decaying exponentials appear in the final linear system.

In compact multiharmonic notation,

$$\underline{H}^{\mathrm{I}}(z) = e^{+jk_{z,\mathrm{inc}}(z-L)}\,\underline{1}_0 + \underline{\underline{e^{-jk_z(z-L)}}}\,\underline{r}, \tag{S97}$$

$$\underline{H}^{\mathrm{TV}}(z) = \underline{\underline{\Psi}}^{\mathrm{TV}}\left(\underline{\underline{e^{-jk_z^{\mathrm{TV}}z}}}\,\underline{a}^{\mathrm{TV}} + \underline{\underline{e^{+jk_z^{\mathrm{TV}}(z-L)}}}\,\underline{b}^{\mathrm{TV}}\right), \tag{S98}$$

$$\underline{H}^{\mathrm{III}}(z) = \underline{\underline{e^{+jk_z z}}}\,\underline{t}. \tag{S99}$$

The tangential electric fields follow from the TM relations. In the air regions,

$$\underline{E}_x^{\mathrm{I}}(z) = \eta\left[-\cos\theta_{\mathrm{inc}}\,e^{+jk_{z,\mathrm{inc}}(z-L)}\,\underline{1}_0 + \underline{\underline{\cos\theta}}\;\underline{\underline{e^{-jk_z(z-L)}}}\,\underline{r}\right], \tag{S100}$$

$$\underline{E}_x^{\mathrm{III}}(z) = -\eta\,\underline{\underline{\cos\theta}}\;\underline{\underline{e^{+jk_z z}}}\,\underline{t}, \tag{S101}$$

and inside the slab, from the multiharmonic constitutive relation in Eq. (S88),

$$\underline{E}_x^{\mathrm{TV}}(z) = \frac{1}{\epsilon_0}\left(\underline{\underline{\omega}}\,\underline{\underline{\epsilon_r}}\right)^{-1}\underline{\underline{\Psi}}^{\mathrm{TV}}\,\underline{\underline{k_z^{\mathrm{TV}}}}\left(\underline{\underline{e^{-jk_z^{\mathrm{TV}}z}}}\,\underline{a}^{\mathrm{TV}} - \underline{\underline{e^{+jk_z^{\mathrm{TV}}(z-L)}}}\,\underline{b}^{\mathrm{TV}}\right). \tag{S102}$$

Enforcing continuity of the tangential fields H_y and E_x at $z = L$ and $z = 0$ for each Floquet harmonic n, and defining the shorthand

$$\underline{\underline{\Phi}} \triangleq \frac{1}{\eta\,\epsilon_0}\left(\underline{\underline{\omega}}\,\underline{\underline{\epsilon_r}}\right)^{-1}\underline{\underline{\Psi}}^{\mathrm{TV}}\,\underline{\underline{k_z^{\mathrm{TV}}}}, \qquad \underline{\underline{P^-}} \triangleq \underline{\underline{e^{-jk_z^{\mathrm{TV}}L}}}, \tag{S103}$$

we obtain the four block equations

$$z = L: \quad \underline{1}_0 + \underline{r} = \underline{\underline{\Psi}}^{\mathrm{TV}}\left(\underline{\underline{P^-}}\,\underline{a}^{\mathrm{TV}} + \underline{b}^{\mathrm{TV}}\right), \tag{S104}$$

$$\cos\theta_{\mathrm{inc}}\,\underline{1}_0 - \underline{\underline{\cos\theta}}\,\underline{r} = \underline{\underline{\Phi}}\left(\underline{b}^{\mathrm{TV}} - \underline{\underline{P^-}}\,\underline{a}^{\mathrm{TV}}\right), \tag{S105}$$

$$z = 0: \quad \underline{\underline{\Psi}}^{\mathrm{TV}}\left(\underline{a}^{\mathrm{TV}} + \underline{\underline{P^-}}\,\underline{b}^{\mathrm{TV}}\right) = \underline{t}, \tag{S106}$$

$$\underline{\underline{\Phi}}\left(\underline{\underline{P^-}}\,\underline{b}^{\mathrm{TV}} - \underline{a}^{\mathrm{TV}}\right) = \underline{\underline{\cos\theta}}\,\underline{t}. \tag{S107}$$

Collecting the unknowns into a single vector, this constitutes the $4(2N{+}1)\times 4(2N{+}1)$ block-matrix system

$$\begin{bmatrix} -\underline{\underline{I}} & \underline{\underline{\Psi}}^{\mathrm{TV}}\underline{\underline{P^-}} & \underline{\underline{\Psi}}^{\mathrm{TV}} & \underline{\underline{0}} \\ \underline{\underline{\cos\theta}} & -\underline{\underline{\Phi}}\,\underline{\underline{P^-}} & \underline{\underline{\Phi}} & \underline{\underline{0}} \\ \underline{\underline{0}} & \underline{\underline{\Psi}}^{\mathrm{TV}} & \underline{\underline{\Psi}}^{\mathrm{TV}}\underline{\underline{P^-}} & -\underline{\underline{I}} \\ \underline{\underline{0}} & -\underline{\underline{\Phi}} & \underline{\underline{\Phi}}\,\underline{\underline{P^-}} & -\underline{\underline{\cos\theta}} \end{bmatrix} \begin{bmatrix} \underline{r} \\ \underline{a}^{\mathrm{TV}} \\ \underline{b}^{\mathrm{TV}} \\ \underline{t} \end{bmatrix} = \begin{bmatrix} \underline{1}_0 \\ \cos\theta_{\mathrm{inc}}\,\underline{1}_0 \\ \underline{0} \\ \underline{0} \end{bmatrix}, \tag{S108}$$

which is solved numerically for the reflection, internal, and transmission harmonic amplitudes. Because only the decaying propagator $\underline{\underline{P^-}}$ appears, the system remains well conditioned even for strongly evanescent slab modes.

Harmonic-resolved observables

The harmonic-resolved reflectance and transmittance follow from the time-averaged Poynting flux in the air regions, normalized to the incident flux:

$$R_n = \frac{\Re\{\cos\theta_n\}}{\cos\theta_{\mathrm{inc}}}\,|r_n|^2, \qquad T_n = \frac{\Re\{\cos\theta_n\}}{\cos\theta_{\mathrm{inc}}}\,|t_n|^2, \tag{S109}$$

which automatically vanish for evanescent orders. The deficit $1-\sum_n(R_n+T_n)$ quantifies the combined effect of Drude absorption and pump–field energy exchange in the modulated slab. In the static limit $M_A = 0$, the operator (S87) becomes diagonal, the eigenvectors reduce to pure harmonics, and r_0 and t_0 recover the standard thin-film (Airy) formulas for a lossy Drude slab at ω_0, which serves as an elementary consistency check.

These quantities map one-to-one onto the outputs of the harmonic-balance FEM solver applied to the same laterally uniform configuration, i.e., the bare modulated ITO layer with air on both sides and neither the TiO_2 grating nor the gold reflector. The comparison therefore constitutes an independent benchmark of the volumetric-polarization implementation of the framework, complementary to the graphene-cylinder benchmark of Sec. 2: it exercises exactly the combination of material dispersion, evaluated at each generated harmonic through Eq. (S87), and modulation-induced inter-harmonic coupling in a bulk Drude medium.

Benchmark of the harmonic-balance framework

The benchmark configuration is shown in Fig. S5(a). A TM-polarized plane wave at $\lambda_0 =$ 1240 nm impinges at the angle $\theta_i = 40^\circ$ on the free-standing modulated ITO slab of thickness L, which generates reflected and transmitted Floquet harmonics with amplitudes r_n and t_n. Because the slab is laterally uniform, all harmonics share the incident transverse wavenumber $k_{x,\mathrm{inc}}$, and each harmonic leaves the slab at its own angle θ_n, as indicated by the rainbow-colored beams in the schematic.

Figure S5(b) compares the harmonic-resolved amplitudes $|r_n|$ and $|t_n|$ obtained from the harmonic-balance FEM simulation with those of the analytical Floquet solution of Eq. (S108), for harmonics $n = -5, \ldots, 5$. The two approaches agree closely for every harmonic, in both reflection and transmission, including the weak higher-order harmonics that lie several orders of magnitude below the carrier. Although the plasma-frequency modulation is single-tone, so that the operator (S87) only couples nearest-neighbor harmonics, the higher-order harmonics are populated through cascaded coupling across the Floquet ladder, and their accurate reproduction confirms that the harmonic-balance system captures this multi-step coupling self-consistently. In addition, the reflected spectrum is not symmetric about $n = 0$: the up- and down-converted harmonics of the same order carry different amplitudes. This asymmetry is consistent with the non-Toeplitz structure of Eq. (S87), in which the Drude response of the ENZ medium is evaluated at the physical frequency of each generated harmonic, and the agreement with theory shows that the harmonic-balance framework reproduces this dispersive feature.

The benchmark also provides a convenient setting for examining the harmonic truncation order, which controls the accuracy of the framework as discussed in the main text. Table S1 lists the convergence criterion of Eq. (15) of the main text, $\left|\mathcal{P}_n^{(N+1)} - \mathcal{P}_n^{(N)}\right| / \left|\mathcal{P}_n^{(N)}\right|$, for the

Table S1: Convergence criterion $\left|\mathcal{P}_n^{(N+1)} - \mathcal{P}_n^{(N)}\right| / \left|\mathcal{P}_n^{(N)}\right|$ of the harmonic powers outgoing from the time-modulated ITO slab (reflected plus transmitted) as a function of the harmonic index n and the truncation order N. Entries with $n > N$ are undefined.

	$N = 2$	$N = 3$	$N = 4$	$N = 5$
$n = 1$	6.05×10^{-3}	5.25×10^{-4}	5.19×10^{-5}	9.94×10^{-6}
$n = 2$	7.18×10^{-2}	5.25×10^{-3}	4.12×10^{-4}	2.69×10^{-5}
$n = 3$	—	3.56×10^{-2}	5.73×10^{-3}	1.14×10^{-4}
$n = 4$	—	—	1.14×10^{-2}	4.60×10^{-3}
$n = 5$	—	—	—	5.07×10^{-3}

harmonic powers $\mathcal{P}_n$ with $n = 1, \ldots, 5$ as the truncation order is increased from $N = 2$ to $N = 5$. Here, $\mathcal{P}_n$ is the total power carried by the n-th harmonic out of the slab, i.e., the sum of the reflected and transmitted powers at ω_n. Only the up-converted harmonics are listed; the down-converted harmonics, $n < 0$, are expected to exhibit similar convergence behavior. Two trends are evident. First, for a fixed harmonic, the relative change decreases monotonically as N increases; for $n = 1$, for example, it falls from 6.05×10^{-3} at $N = 2$ to 9.94×10^{-6} at $N = 5$. Second, for a fixed truncation order, the relative change is largest for the outermost retained harmonics, $n \approx N$, which are most directly affected by the omission of harmonics beyond the retained window, whereas the low-order harmonics carrying most of the frequency-converted power converge rapidly. At $N = 5$, the relative change is below 1.2×10^{-4} for $n \leq 3$ and below 5.1×10^{-3} for all listed harmonics, so that every harmonic power varies by less than 1% when an additional harmonic is added to each side of the Floquet basis. This rapid convergence reflects the nearest-neighbor coupling produced by the single-tone modulation, through which the power reaching the n-th harmonic decreases quickly with $|n|$, and it confirms that the retained harmonic set provides an accurate representation of the steady-state response. Because the relative change of every harmonic power continues to decrease with N, a smaller tolerance η in Eq. (15) of the main text can be satisfied simply by retaining a larger number of harmonics, at the cost of a proportionally larger harmonic-balance system.

This simple example therefore serves as a benchmark demonstrating that the framework accurately simulates dispersive time-varying bulk materials. Together with the graphene-cylinder benchmark for time-varying sheets, it validates both secondary-source representations of the formulation, namely the surface current densities of modulated sheets and the volumetric polarization densities of modulated bulk media.

6 Shape Optimization of the ITO-Based Space–Time Metasurface

The supercell obtained from the unit-cell phase library provides only an approximate realization of the desired space–time response, because it neglects near-field coupling between adjacent grating blocks, the dispersive epsilon-near-zero response of the modulated ITO layer, and the inter-harmonic coupling that redistributes power among the generated sidebands.

To refine the initial design, we apply a gradient-based shape optimization directly to the full harmonic-balance model, so that all retained Floquet harmonics are solved simultaneously at every design iteration.

The design freedom is restricted to the TiO_2 grating layer. Each of the three dielectric blocks in the supercell is allowed to translate along x and to be scaled in width, while its thickness, the ITO layer, the gold reflector, and the supercell period Λ remain fixed. This yields a compact design vector $\boldsymbol{\xi} \in \mathbb{R}^6$ containing the lateral position and width of each block. Bounds are imposed on the translations and widths so that neighboring blocks cannot overlap or cross the periodic boundary. Rather than regenerating the geometry at each iteration, the design update is applied as a smooth deformation of the computational mesh, which keeps the mesh connectivity fixed and allows the design sensitivities to be evaluated analytically.

The device targets the frequency-converted first spatial diffraction order in reflection. Let $H^{\text{refl}}_{m,n}$ denote the complex amplitude of the reflected magnetic field projected onto the Floquet–Bloch channel with spatial order m and temporal harmonic n, obtained by overlap integration of the harmonic-balance solution on the port plane. The optimization maximizes the power carried by the two targeted sideband channels,

$$Q(\boldsymbol{\xi}) = \sum_{n=\pm 1} \frac{\left|H^{\text{refl}}_{1,n}\right|^2}{\left|H^{\text{inc}}\right|^2}, \tag{S110}$$

where H^{inc} is the incident magnetic-field amplitude. The objective is formulated in terms of the squared modulus rather than the modulus itself, since the former is a smooth function of the complex field unknowns and therefore admits well-defined partial derivatives; maximizing either quantity yields the same optimal design. Note that Q deliberately rewards both the upper and lower sidebands, $n = +1$ and $n = -1$, at the same spatial order $m = 1$, consistent with the frequency-converting beam-deflection functionality described in the main text.

Here $H^{\text{refl}}_{1,n}$ and H^{inc} are the complex magnetic-field amplitudes of the corresponding Floquet–Bloch channels. The fraction of the incident power carried by a propagating reflected channel follows by weighting the squared amplitude ratio with the obliquity factor $\Re\{\cos\theta_{m,n}\}/\cos\theta_{\text{inc}}$ [cf. Eq. (S109)], where $\theta_{m,n}$ is the propagation angle of the channel. Because the supercell period and the harmonic frequencies are fixed, the angles of the target channels, $\theta_{1,+1} \approx 53.4^\circ$ and $\theta_{1,-1} \approx 54.9^\circ$, do not change during the optimization; the obliquity factors are therefore constant positive weights, and maximizing Q is equivalent to maximizing the power converted into the target channels. In terms of power, the optimized design converts approximately 1.2% and 1.4% of the incident power into the $(m, n) = (1, +1)$ and $(1, -1)$ channels, respectively (a total of ≈2.6%), up from approximately 0.8% and 1.0% for the initial phase-library design. These conversion efficiencies are set primarily by the modulation depth M_A and by the dissipation in the ENZ ITO layer, and are to be distinguished from the directional redistribution among the spatial orders that the optimization targets.

Writing the discretized harmonic-balance system as $\mathcal{L}(\underline{\mathbf{X}}, \boldsymbol{\xi}) = \mathbf{0}$, with $\underline{\mathbf{X}}$ the multi-harmonic field vector and $\mathbf{J} = \partial\mathcal{L}/\partial\underline{\mathbf{X}}$ the system Jacobian, the gradient of the objective is obtained by the adjoint method,

$$\mathbf{J}^{\mathsf{T}}\,\underline{\mathbf{X}}^{*} = \left(\frac{\partial Q}{\partial \underline{\mathbf{X}}}\right)^{\mathsf{T}}, \qquad \frac{\mathrm{d}Q}{\mathrm{d}\boldsymbol{\xi}} = \frac{\partial Q}{\partial \boldsymbol{\xi}} - \left(\underline{\mathbf{X}}^{*}\right)^{\mathsf{T}} \frac{\partial \mathcal{L}}{\partial \boldsymbol{\xi}}. \tag{S111}$$

The key practical point is that the finite-element framework assembles and factorizes $\mathbf{J}$ during the forward harmonic-balance solve. The adjoint field $\underline{\mathbf{X}}^*$ is therefore recovered by a single back-substitution with the transposed factor, and the full gradient with respect to all design variables follows at negligible additional cost. Unlike explicit time-domain solvers, which require a separate reverse-time simulation to construct the adjoint field, no second electromagnetic simulation is needed here. Because the modulated ITO layer couples the harmonics through the time-dependent Drude response, the adjoint solve automatically accounts for the sensitivity of every retained harmonic to a perturbation of the grating geometry. The design is updated using a globally convergent variant of the method of moving asymptotes, which is well suited to smooth, bound-constrained problems with a small number of design variables, and a per-iteration move limit is enforced to prevent excessive distortion of the deforming mesh. Starting from the phase-library supercell, the optimization is run for 50 iterations.

In these simulations, the fields are discretized with quadratic Lagrange elements, with a maximum element size of $0.02\lambda_0$ inside the time-varying ITO layer and a mesh that coarsens gradually away from it; the fully coupled multiharmonic system for $N = 4$ comprises 341,244 degrees of freedom and is factorized and solved by a direct sparse solver in about 11 s (roughly 19 s including assembly) on a ten-core Intel Xeon workstation, so that each design iteration of the shape optimization costs well under a minute.

In Fig. S6(a), the normalized reflected amplitudes $|H_{1,n}^{\mathrm{refl}}|/|H^{\mathrm{inc}}|$ of both sidebands increase monotonically, apart from small oscillations in the early iterations. The upper sideband ($n = 1$) rises from approximately 0.112 to 0.142 and the lower sideband ($n = -1$) from approximately 0.130 to 0.153, corresponding to relative improvements of roughly 27% and 18%, respectively. Both curves plateau after about 25–30 iterations, indicating that the design has converged to a local optimum within the imposed bounds. That both channels improve simultaneously confirms that the two sidebands are not competing for the same power budget; instead, the optimizer redistributes power away from the untargeted $m = 0$ and $m = -1$ channels.

The corresponding geometric changes are modest. Measured from the center of the supercell, the initial phase-library design places the three blocks at x-centers of -760.0, -110.0, and 540.0 nm, with widths of 78.0, 364.0, and 526.5 nm, equivalent to filling factors of 0.12, 0.56, and 0.81 of the 650 nm unit cell. After 50 iterations the blocks are centered at -789.6, -156.8, and 590.0 nm, with widths of 62.4, 413.6, and 551.7 nm, corresponding to filling factors of 0.096, 0.636, and 0.849. The narrow first block is thus made narrower still and displaced outward, whereas the two wider blocks are broadened, and no block is displaced by more than 50 nm, i.e. less than 3% of the supercell period. Most of this rearrangement occurs early: by iteration 10 the blocks have already reached widths of 62.4, 428.7, and 547.6 nm and centers of -757.2, -128.9, and 559.2 nm, so that the remaining forty iterations refine the design only slightly while the targeted amplitudes in Fig. S6(a) continue to grow. This indicates that the performance gain originates from fine adjustment of the inter-block near-field coupling rather than from a gross redistribution of the dielectric material, and it justifies treating the phase-library supercell as a good initial guess.

Figures S6(b) and S6(c) show the magnetic-field distributions H_z at the lower and upper sideband frequencies, ω_{-1} and ω_1, for these three representative iterations. At iteration 0 the sideband fields are weak and largely localized within the dielectric blocks, reflecting

the fact that the phase-library supercell was assembled from isolated unit-cell responses. As the optimization proceeds, the block positions and widths adjust so that the sideband fields extend into the ENZ region and acquire the transverse phase progression required for deflection into the $m = 1$ order. By iteration 50, both sidebands display the tilted wavefronts characteristic of the intended frequency-converting deflection, in agreement with the far-field diffraction angles reported in the main text.

7 Three-dimensional space–time metasurface with subwavelength TiO_2 disks on modulated ITO

Both examples in the main text are two-dimensional: the rolled graphene cylinder is invariant along its axis, and the reflective metasurface is uniform along y. The harmonic-balance formulation itself, however, places no restriction on the dimensionality of the problem. Each harmonic subproblem in Eq. (13) of the main text is a standard frequency-domain vector finite-element problem, and the modulation enters only through the secondary-source coupling operators. Here, we apply the framework to a fully three-dimensional configuration, closely related to the reflective metasurface of the main text, in which the one-dimensional binary grating is replaced by a two-dimensional array of TiO_2 disks.

The structure is shown in Fig. S7(a). A square array of TiO_2 disks with radius R and period $P = 950$ nm along both x and y is placed on a time-modulated ITO layer backed by a gold reflector. The disk height, the ITO thickness, the gold reflector, and the ITO material model are identical to those of the metasurface in the main text. In particular, the ITO is described by the same Drude response operating near its epsilon-near-zero wavelength, $\lambda_0 = 1240$ nm, and its squared plasma frequency is modulated as $A(t) = A_0\,[1 + M_A \cos(\Omega t + \alpha)]$ with $\Omega/2\pi = 3$ THz and $M_A \simeq 0.105$. The volumetric secondary polarization of the ITO therefore follows Eq. (18) of the main text, with the Drude denominator evaluated at each harmonic frequency ω_n. The array is illuminated by a TM-polarized plane wave, with the magnetic field along y, incident in the x–z plane at $\theta_{\text{inc}} = 10°$, the same angle as in the main text. A single unit cell is simulated with Floquet-periodic boundary conditions along x and y, and the reflected field of each harmonic is projected onto the corresponding Floquet–Bloch channels.

In contrast to the supercell of the main text, the period here is subwavelength, $P < \lambda_0$. For the reflected space–time orders, the transverse wavenumbers $k_0 \sin\theta_{\text{inc}} + 2\pi m_x/P$ and $2\pi m_y/P$ exceed the free-space wavenumber $k_n = \omega_n/c$ of every retained harmonic whenever $(m_x, m_y) \neq (0, 0)$, because $\Omega/\omega_0 \approx 0.012$ shifts the harmonic frequencies only slightly from the carrier. Consequently, only the spatial zeroth order, $m = 0$, propagates, and the array redistributes the incident power solely among the temporal harmonics of the specular channel, $H^{\text{refl}}_{0,n}$. The device therefore acts as a frequency-converting mirror rather than a beam deflector, and the relevant design question is how the disk geometry controls the partition of the reflected power between the carrier and the generated sidebands.

Figure S7(b) shows the normalized reflected amplitudes $|H^{\text{refl}}_{0,n}|/|H^{\text{inc}}|$ of the carrier, $n = 0$, and of the first sidebands, $n = \pm 1$, as functions of the disk radius. As the radius increases, the disks approach a localized resonance of the TiO_2-disk/ITO/Au stack and the carrier

reflection drops sharply, with strong variations as resonant features of the array shift through the operating wavelength. Over the same range, both sideband amplitudes increase. For $R = 400$ nm, the carrier amplitude falls below the amplitudes of both first-order sidebands, so that the reflected power is dominated by the frequency-converted channels.

The complete harmonic-resolved specular response for $R = 400$ nm is shown in Fig. S7(c). The $n = \pm 1$ sidebands carry the largest amplitudes, followed by the suppressed carrier, while the $n = \pm 2$ and $n = \pm 3$ harmonics decay progressively with increasing harmonic order. Because the modulation is single-tone, the second- and third-order harmonics are not generated directly by the modulation spectrum. They arise from cascaded coupling through the nearest-neighbor off-diagonal blocks of the harmonic-balance system, and their presence shows that the multi-harmonic system is solved self-consistently rather than in a first-order perturbative manner. The nearly symmetric distribution about $n = 0$ reflects the small ratio Ω/ω_0; the residual asymmetry originates from the ENZ dispersion of ITO, which is evaluated at the physical frequency of each harmonic.

Figures S7(d)–S7(f) show the magnetic-field distributions H_y within one unit cell for $n = -1$, 0, and 1, respectively, at $R = 400$ nm. At the carrier frequency, the field is strongly concentrated in the TiO_2 disk and reaches amplitudes substantially larger than those of the sidebands. This behavior is consistent with the resonant suppression of the carrier reflection in Fig. S7(b): the incident energy is trapped by the localized resonance, where it is either dissipated in the lossy ENZ layer or converted into sidebands, rather than being specularly reflected. The enhanced carrier near field, in turn, strengthens the modulation-induced polarization in the underlying ITO layer. The sideband fields in Figs. S7(d) and S7(f) retain the spatial structure of the resonant carrier field within the disk, and they also extend into the modulated ITO layer, where the secondary polarization sources responsible for their generation reside.

This example shows that the harmonic-balance framework carries over directly to three-dimensional dispersive time-varying structures. Each harmonic subproblem retains the full three-dimensional vector finite-element discretization of the disk array, and the number of unknowns grows by the factor $2N + 1$ relative to a single-frequency simulation, as discussed in the main text. The time-varying ITO layer is still represented by the same volumetric secondary polarization, with no modification of the formulation. Combined with geometric parameter sweeps such as the one in Fig. S7(b), or with gradient-based optimization of the type described in Sec. 6, the framework therefore enables the design of three-dimensional space–time metasurfaces with realistic material dispersion.

References

(S1) Galiffi, E. *et al.* Wood anomalies and surface-wave excitation with a time grating. *Physical Review Letters* **125**, 127403 (2020).

(S2) Hanson, G. W. Dyadic green's functions and guided surface waves for a surface conductivity model of graphene. *Journal of Applied Physics* **103** (2008).

(S3) Falkovsky, L. A. & Varlamov, A. A. Space-time dispersion of graphene conductivity. *The European Physical Journal B* **56**, 281–284 (2007).

(S4) Shirokova, A. V., Maslov, A. V. & Bakunov, M. I. Surface plasmon transformation on dynamic graphene with a periodic modulation of carrier density. *Physical Review B* **108**, 245139 (2023).

(S5) Balanis, C. A. *Advanced engineering electromagnetics* (John Wiley & Sons, 2012).

(S6) Tavakol, M. R. & Cai, W. Nonreciprocal negative refraction enabled by photonic time crystals. *Nano Letters* **26**, 1569–1577 (2026).

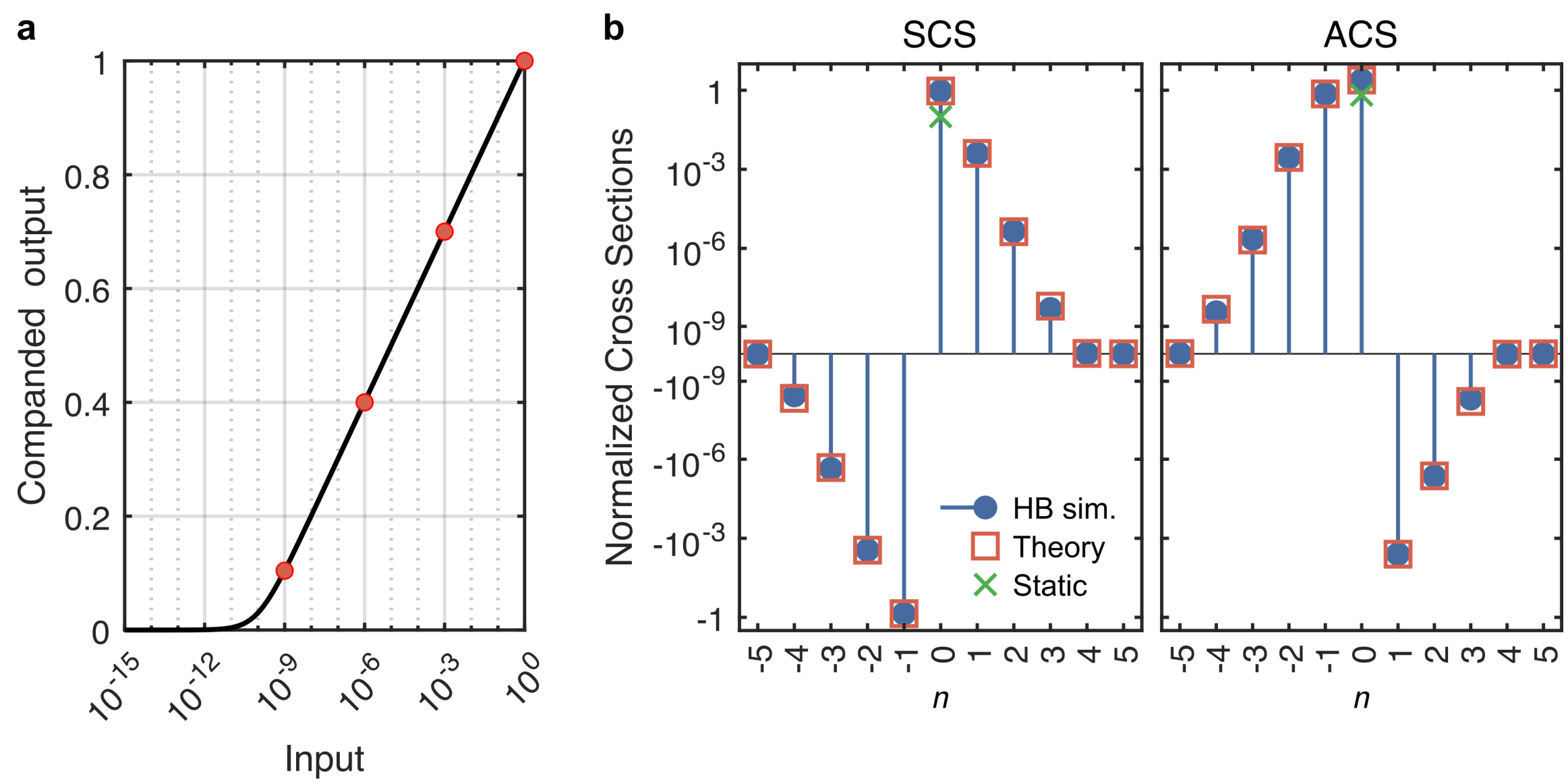

Figure S1: **Companded harmonic-resolved response of the parametrically pumped rolled graphene cylinder.** (a) Positive-input μ-law compander characteristic for $\mu = 10^{10}$, used to map cross-section values spanning many decades onto a visually resolved nonlinear scale. The red markers correspond to the original values used as vertical-axis ticks in panel (b). (b) Normalized scattering and absorption cross sections resolved over Floquet harmonics and plotted on the companded scale. The tick labels indicate the original uncompanded values, making weak harmonic responses down to $\sim 10^{-10}$ distinguishable. The harmonic-balance simulation and analytical theory remain in excellent agreement across all resolved harmonics, with the static response shown for reference.

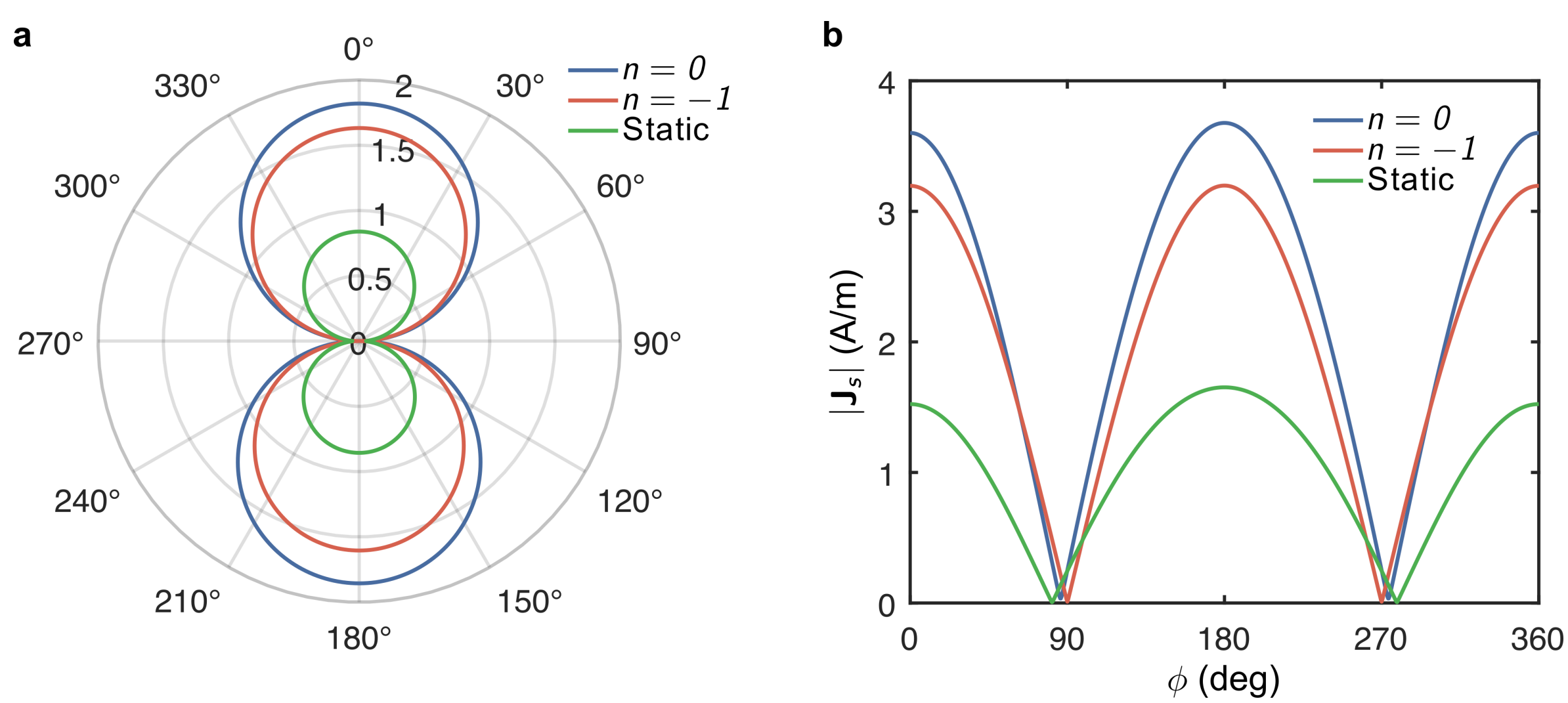

Figure S2: **Far-field radiation and surface-current profiles of the parametrically pumped rolled graphene cylinder.** (a) Far-field electric-field pattern, $\mathbf{E}^{\text{far}}(\phi)$, for the static cylinder and for the $n = 0$ and $n = -1$ Floquet harmonics of the parametrically pumped graphene cylinder. (b) Graphene surface-current density versus polar angle ϕ for the same cases, comparing the static response with the carrier and down-converted harmonic currents generated by the temporal modulation.

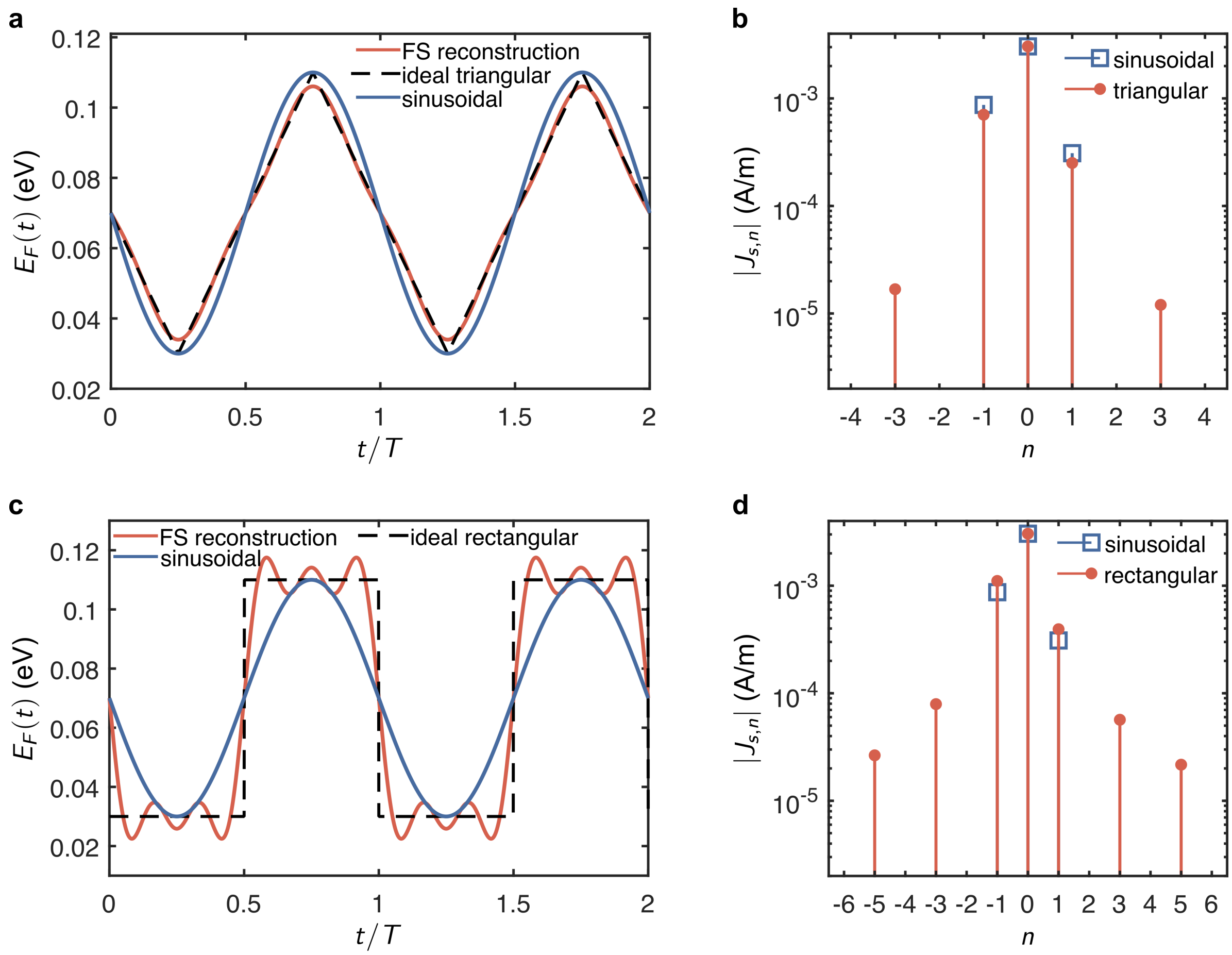

Figure S3: **Non-sinusoidal Fermi-level modulation of the parametrically pumped rolled graphene cylinder.** (a) Triangular Fermi-level modulation profile, $E_F(t)$, of the rolled graphene sheet. The ideal triangular waveform is compared with its truncated Fourier-series (FS) reconstruction retaining three modulation harmonics, shown in red, and with the sinusoidal profile of Eq. (16) in the main text. All profiles share $E_{F0} = 70$ meV and $\Delta E_F = 40$ meV. (b) Magnitudes of the FS coefficients, $|J_{s,n}|$, of the steady-state surface current density $J_s(t)$ induced by a normally incident electric field with $E_0 = 1$ V/m and $f_0 = 0.4$ THz under the triangular modulation profile in panel (a). The corresponding coefficients for sinusoidal modulation are shown for reference. (c) Rectangular Fermi-level modulation profile, $E_F(t)$, of the rolled graphene sheet, compared with its truncated FS reconstruction retaining five modulation harmonics, shown in red, and with the sinusoidal profile of Eq. (16). (d) Magnitudes of the FS coefficients, $|J_{s,n}|$, of the steady-state surface current density under the rectangular modulation profile in panel (c), with the sinusoidal case shown for reference. Owing to the half-wave symmetry of both non-sinusoidal profiles, only odd-order sidebands are generated, and the number of significant sidebands grows with the harmonic content of the modulation waveform. Time in panels (a,c) is normalized to the modulation period T.

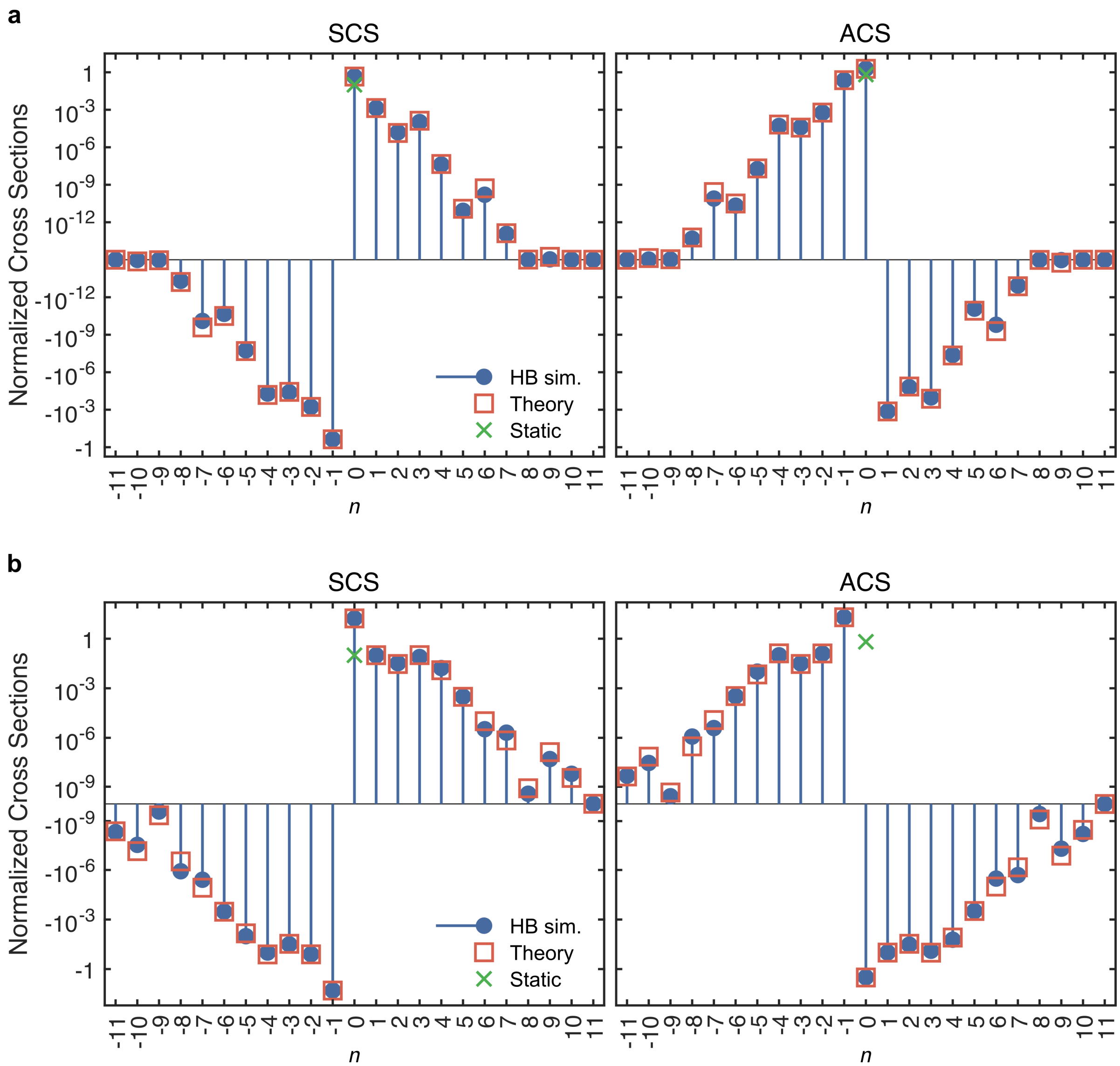

Figure S4: **Companded harmonic-resolved response of the rolled graphene cylinder under non-sinusoidal Fermi-level modulation.** (a) Normalized scattering and absorption cross sections resolved over Floquet harmonics for the triangular modulation profile in Fig. S3(a), plotted on a μ-law companded scale with $\mu = 10^{15}$. (b) Same quantities for the rectangular modulation profile in Fig. S3(c), plotted on a μ-law companded scale with $\mu = 10^{10}$. The tick labels indicate the original uncompanded values, making weak harmonic responses down to $\sim 10^{-12}$ in panel (a) and $\sim 10^{-9}$ in panel (b) distinguishable. The harmonic-balance simulation and analytical theory are in excellent agreement across all resolved harmonics for both modulation profiles, with the static response shown for reference.

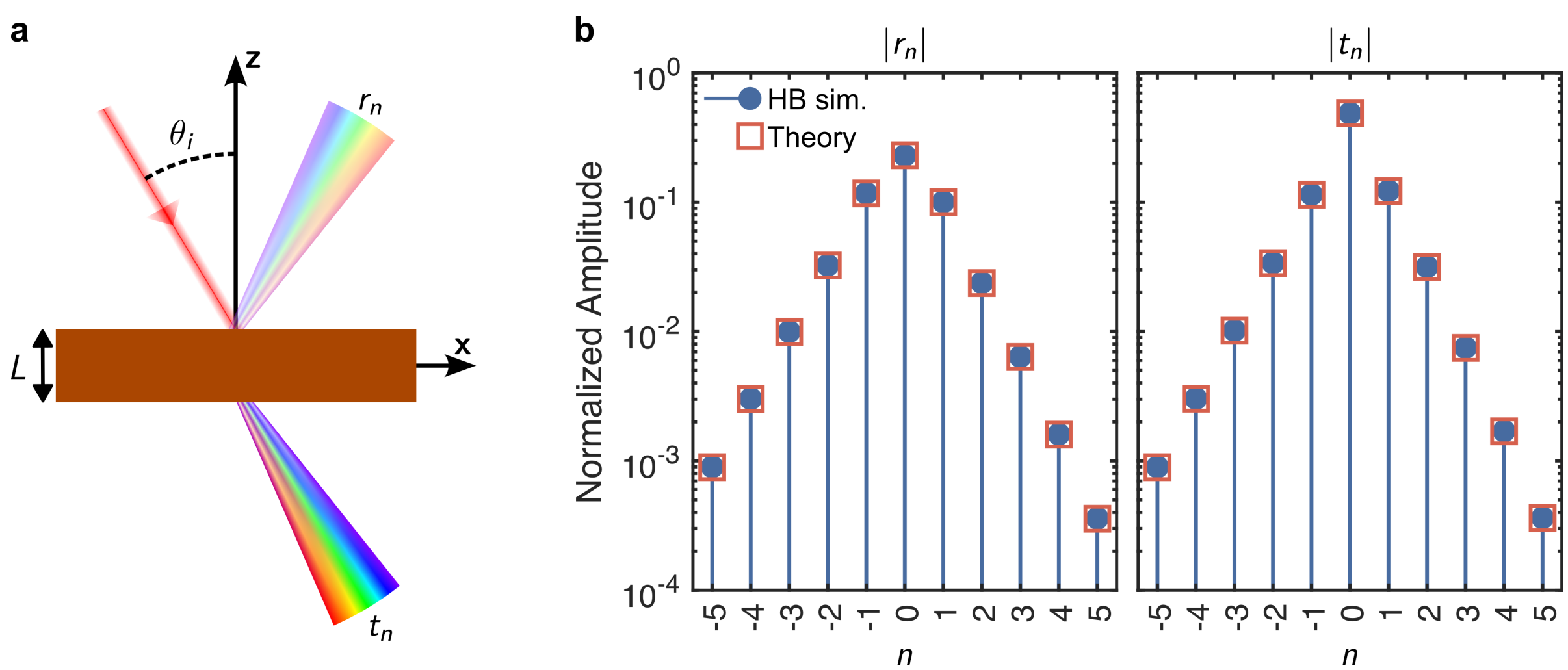

Figure S5: **Benchmark of the harmonic-balance framework for a time-modulated dispersive ITO slab.** (a) Schematic of a free-standing ITO slab of thickness $L = 300$ nm with temporally modulated plasma frequency, illuminated by a TM-polarized plane wave at $\lambda_0 = 1240$ nm and incidence angle $\theta_i = 40°$. The reflected and transmitted fields consist of Floquet harmonics with amplitudes r_n and t_n. (b) Harmonic-resolved normalized reflection and transmission amplitudes, $|r_n|$ and $|t_n|$, obtained from the harmonic-balance (HB) simulation and the analytical Floquet solution.

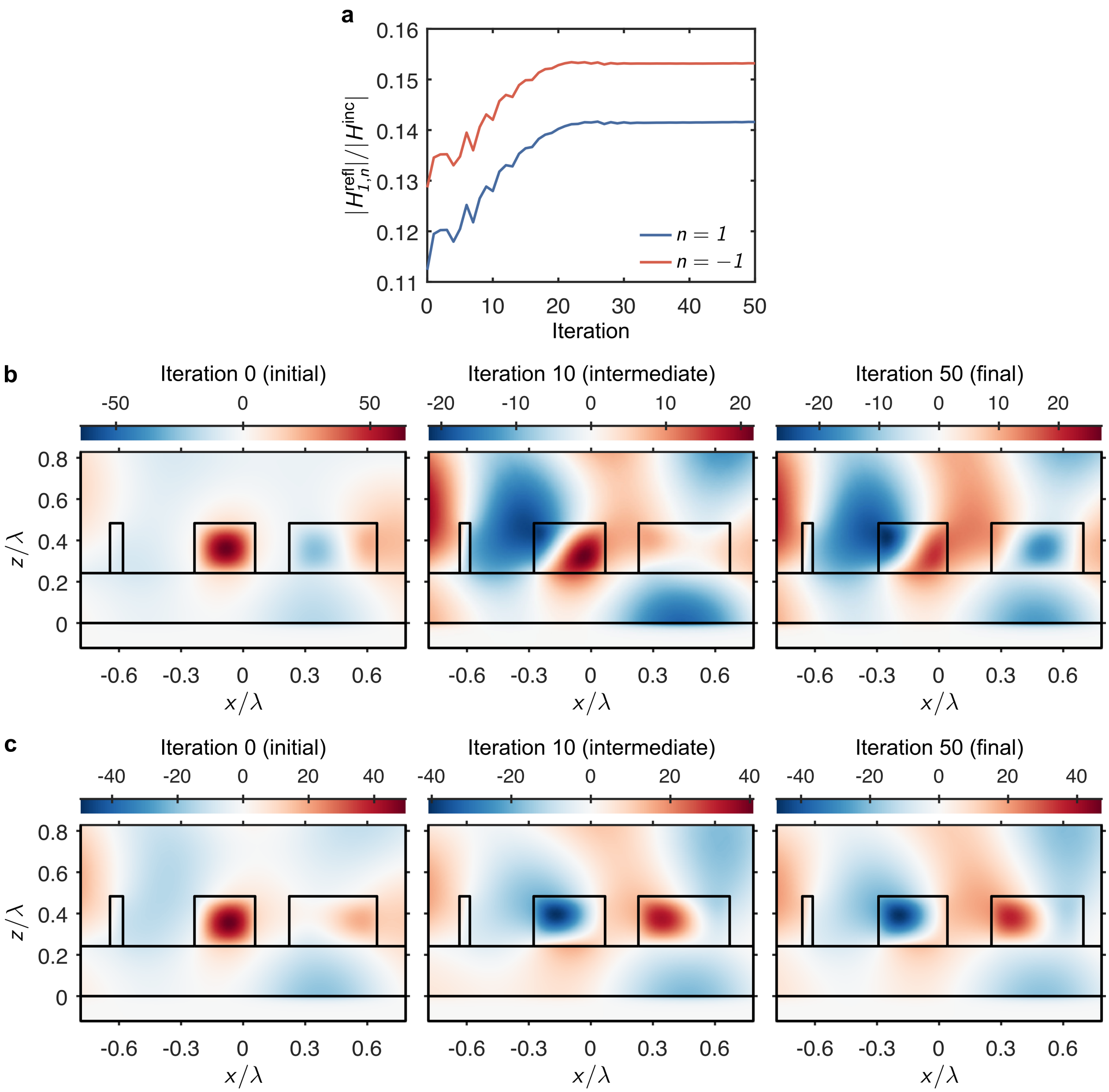

Figure S6: **Evolution of the targeted space–time diffraction channels during shape optimization.** (a) Normalized reflected amplitudes of the $m = 1$ spatial diffraction channel at the lower and upper sideband harmonics, $n = -1$ and $n = 1$, as a function of shape-optimization iteration. Both targeted channels increase during the optimization process. (b,c) Magnetic-field distributions, H_z, at representative optimization iterations for the lower sideband, ω_{-1}, and upper sideband, ω_1, respectively, under an incident power of 1 W/m over one supercell period. The field profiles show the gradual formation of the desired reflected diffraction response from the initial geometry to the final optimized metasurface. Color bars in panels (b,c) are in units of A/m.

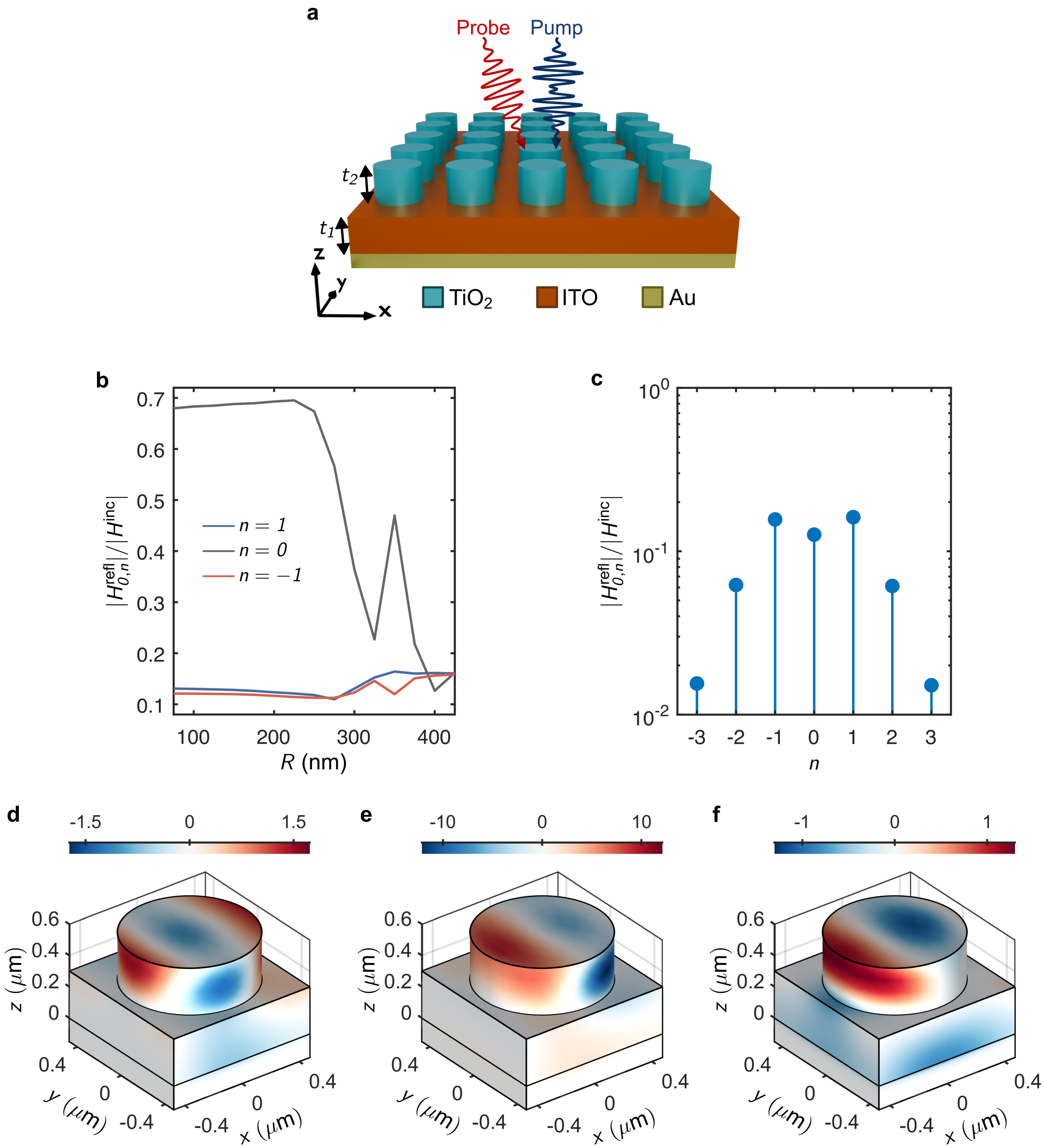

Figure S7: **Three-dimensional space–time metasurface with subwavelength TiO_2 disks on modulated ITO.** (a) Schematic of the TiO_2 disk array on a time-varying ITO layer backed by a gold reflector, with ITO and TiO_2 thicknesses $t_1 = t_2 = 300$ nm. (b) Normalized zeroth-order reflected amplitudes of the harmonics $n = -1$, 0, and 1 versus disk radius R. (c) Harmonic-resolved normalized zeroth-order reflected amplitudes for $R = 400$ nm. (d–f) Magnetic-field distributions, H_y, for $R = 400$ nm at (d) $n = -1$, (e) $n = 0$, and (f) $n = 1$, under an excitation power of 1 nW per unit cell. Field maps are in A/m.
